# Association between accelerometer-measured physical activity and mortality in cancer survivors: A prospective cohort study from UK Biobank

**DOI:** 10.1016/j.jnha.2025.100586

**Published:** 2025-05-26

**Authors:** Zhihan Jiang, Bingyan Wang, Yifei Zhao, Jing Weng, Jiaojiao Liao, Liyuan Tao, Kui Sun, Zhipeng Zhang, Xin Zhou, Wei Fu

**Affiliations:** a Department of General Surgery, Peking University Third Hospital, Peking University, Beijing 100191, China; b Beijing Key Laboratory for Interdisciplinary Research in Gastrointestinal Oncology (BLGO), Beijing 100191, China; c Clinical Epidemiology Research Center, Peking University Third Hospital, Peking University, Beijing 100191, China; d Peking University Third Hospital Cancer Center, Peking University, Beijing 100191, China

**Keywords:** Physical activity, Accelerometer, Cancer survivors, Mortality, Dose-response association

## Abstract

**Objectives:**

Postdiagnosis physical activity is an important component of healthy lifestyle in cancer survivors. In this study, we aimed to explore the association between intensity and duration of physical activity measured by wearable accelerometers and mortality among pan-cancer survivors.

**Methods:**

A prospective cohort study involving cancer survivors (*n* = 11,708) from UK Biobank was performed. All participants had thorough physical activity data that was measured by wrist-worn accelerometers. Restricted cubic splines and multivariate Cox proportional hazards models were employed to assess the dose-response associations between physical activity time at varying intensities and both all-cause and cancer-specific mortality.

**Results:**

During a median follow-up of 8.9 years, a total of 983 deaths occurred, including 656 cancer-related deaths. Multivariate models identified significant dose-response associations between moderate to vigorous-intensity physical activity (MVPA) time and mortality. Hazard ratios (HRs) for all-cause mortality were 0.64 (95% CI, 0.54–0.76), 0.61 (95% CI, 0.51–0.74) and 0.52 (95% CI, 0.42–0.66) in participants with MVPA time of 272–407, 407–579 and ≥579 min per week, respectively. HRs for cancer-specific mortality were 0.71 (95% CI, 0.58–0.88), 0.69 (95%CI, 0.55–0.87) and 0.61 (95%CI, 0.47–0.81) for the aforementioned groups. Similar patterns were observed for moderate-intensity physical activity but not for light-intensity physical activity. Survival benefits of active physical activity were pronounced in cancers from multiple organs.

**Conclusions:**

Active physical activity substantially reduced all-cause mortality in pan-cancer survivors and cancer-specific mortality in cancer survivors of specific sites. However, the benefits were significant only when intensity of physical activity reached moderate to vigorous level.

## 1 Introduction

Cancer remains the major cause of death globally, and its burden is projected to continue rising in coming decades [1,2]. Nearly 20.0 million new cancer cases and 9.7 million cancer deaths were reported in 2022 [3]. Recent developments in cancer treatments have improved the prognosis of certain types of cancer [4]. Nevertheless, the overall 5-year survival rate for all cancers is around 68% and cancer survivors often suffer from cancer-related or treatment-derived side effects, posing ongoing challenges for long-term management of cancer patients [5].

Physical activity has emerged as a modifiable factor associated with the incidence and mortality of cancer. Mediated by multiple mechanisms, physical activity is linked to significantly lower the risk of cancers and cancer-specific mortality in the general population [[6], [7], [8], [9]]. Several studies have focused on survival benefit of physical activity in cancer survivors. A meta-analysis found that the highest amount of postdiagnosis physical activity reduced hazards of cancer-specific mortality by 37% in cancer survivors, and this effect was observed in breast cancer, colorectal cancer and prostate cancer subgroups [10]. Four recent observational studies showed that sufficient physical activity reduced risks of both all-cause mortality and cancer-specific mortality [[11], [12], [13], [14]]. However, data on physical activity was primarily obtained using questionnaires, which were susceptible to recall bias inevitably [12]. Another study leveraged a cohort of 480 participants with accelerometry-derived data, whereas, subgroup analyses in different types were not sufficiently robust due to the small sample size [15].

To address aforementioned gaps, we aimed to comprehensively investigate the association between intensity and duration of physical activity and all-cause mortality as well as cancer-specific mortality in cancer survivors from the UK Biobank prospective cohort, comprising more than 100,000 participants with physical activity data measured using wrist-worn accelerometers. Besides the dose-response associations between physical activity and survival, we also explored the associations in cancers from separate organ systems, aiming to optimize benefits among all cancer survivors.

## 2 Material and methods

### 2.1 Population and study design

The UK Biobank is a national prospective cohort, in which more than 0.5 million participants were recruited to complete a touch-screen questionnaire and other examinations for baseline evaluation between April 2007 and December 2010 [16]. Participants provided informed consent and ethical approval was provided by the UK National Health Service (NHS), National Research Ethics Service (Ref 11/NW/0382). All participants in this study were from UK Biobank (Application: 107479), and detailed eligibility criteria were provided in Supplementary Methods. Flowchart of participant inclusion and exclusion was shown in Supplementary Fig. S1. According to International Classification of Diseases, 10th Revision (ICD-10, C00-C97, D00-D09, D10-D36, and D37-D48), 119,326 participants with cancer registry records were initially included. We further excluded participants without valid accelerometer-measured physical activity data (*N* = 97,612), participants whose first cancer diagnosis occurred after wearing accelerometers (*N* = 8710), and those with missing covariates values (*N* = 1296). Final analysis included 11,708 participants, and the case numbers of different cancer types are presented in Supplementary Table S1, considering multiple cancer sites in each individual.

### 2.2 Accelerometer-measured physical activity

Participants in UK Biobank were invited to wear an accelerometer for seven days from February 2013 to December 2015, with 103,617 participants having complete data [17]. The sensor in accelerometer captured the triaxial acceleration at 100 Hz with a dynamic range of ±8 *g* (unit of gravity) [18]. The accelerometer recorded the time of activity under different accelerations, which was converted into intensity and duration of physical activity. Duration of light-intensity physical activity (LPA), moderate-intensity physical activity (MPA), and vigorous-intensity physical activity (VPA) corresponded to the time spend in 30–125 milligravities (mg), >125–400 mg, and >400 mg intensity activity, respectively [19,20]. Duration of moderate to vigorous-intensity physical activity (MVPA) was calculated as sum of MPA and VPA. More details regarding physical activity were provided in Supplementary Methods.

### 2.3 Outcomes assessment

Primary outcomes of this study were all-cause mortality and cancer-specific mortality. Mortality records were linked to the UK Biobank dataset from NHS Digital (England and Wales) and from the Information and Statistics Division (Scotland). The censoring dates were 30 November 2023 and 31 December 2023, respectively. Follow-up duration was defined as the interval between date of wearing accelerometer and date of death occurrence or censoring date, whichever occurred first. Causes of death were determined using ICD-10 coding, and mortality was deemed to be cancer-specific if the underlying primary cause of death was attributed to cancer (codes C00-C96).

### 2.4 Covariates ascertainment

Potential covariates were selected by reviewing prior researches and leveraging knowledge concerning the relationship between lifestyle and the prognosis of cancer survivors [21,22]. Additionally, factors which might influence the measurement of physical activity were also selected and evaluated. All variables included age (years between birth date and start of wearing accelerometer), gender (male or female), Townsend deprivation score, ethnicity (White, Asian, Blacks, mixed, or other), smoking status (current smoker, former smoker, or never), alcohol intake frequency (daily, 3–4 times/week, 1–2 times/week, 1–3 times/month, social drinking or never), body mass index (BMI, kg/m^2^, calculated based on the ratio of weight in kilograms to height in meters squared), waist circumference (cm), self-reported general health (excellent, good, fair, or poor), cancer duration (years between first cancer diagnosis date and start of wearing accelerometer), cancer biological behavior (defined as malignant if cancer diagnosis in C00-C97), cardiovascular disease history (including heart attack, angina, stroke or hypertension), diabetes history, long-standing illness, disability or infirmity, grip strength (kg, defined as average grip strength of both hands), forced expiratory volume in 1 second (FEV1, filter) and wear season (based on start of wearing accelerometer and classified as spring, summer, autumn, or winter).

### 2.5 Statistical analysis

Baseline characteristics of participants were described by the mean ± standard deviation (SD) or median [interquartile range, IQR] for continuous variables and number and percentage for categorical variables according to the quartiles of MVPA time (minutes/week). Analysis of variance (ANOVA) or Kruskal-Wallis test (for cancer duration) and χ^2^ test were applied, as appropriate.

The correlation between LPA time and MVPA time was represented by Pearson coefficients. Dose-response associations between physical activity duration and outcomes were evaluated using Cox proportional hazards models and restricted cubic splines (RCS), with number of knots setting as three. The proportional hazards assumption was evaluated using Schoenfeld residual plots. Departure from linearity was assessed based on Wald tests, with a P value <0.05 indicating significant non-linearity. RCS that analyzed dose-response association between LPA time and outcomes were adjusted for MVPA time (continuous variable, minutes), age (continuous variable, years), gender, Townsend deprivation score, ethnicity, smoking status, alcohol intake frequency, BMI (continuous variable, kg/m^2^), waist circumference (continuous variable, cm), reported general health, cancer duration (continuous variable, years), cardiovascular disease history, diabetes history, long-standing illness, disability or infirmity, grip strength, FEV1, and wear season. Likewise, RCS that analyzed dose-response association between MVPA time and outcomes were adjusted for LPA time (continuous variable, minutes) and the other covariates, considering the high correlation between LPA time and MVPA time. Furthermore, duration of physical activity of various intensities was categorized into four levels based on quantiles and recommended values from current guideline: <272, 272–407, 407–579, and ≥579 min/week for MVPA, <1637, 1637–1939, 1939–2242, and ≥2242 min/week for LPA, and <150, 150–300, 300–450, and ≥450 min/week for MPA (according to current guideline recommendations) [23]. Multivariate Cox proportional hazards models were performed to estimate the hazard ratio (HR) and 95% confidence intervals (CIs) for the association of LPA, MVPA, and MPA with all-cause and cancer-specific mortality. The same covariates as mentioned above were adjusted. To examine the independent association, LPA, MVPA, and MPA were adjusted for each other in all models. Linear trends were examined using the median value of each physical activity category as a continuous variable into the models [24].

Subgroup analyses were performed for all-cause and cancer-specific mortality stratified by age (<65 and ≥65 years old), sex (female and male), BMI (<30 and ≥30 kg/m^2^), waist circumference (unhealth and health), cancer biological behavior (malignant and non-malignant), grip strength (low and high), FEV1 (<2.63 and ≥2.63 L) and cancer duration (<5 and ≥5 years). HRs and 95% CIs were calculated by comparing participants with MVPA ≥ 272 min/week (or LPA ≥ 1637 min/week) to those with MVPA < 272 min/week (or LPA < 1637 min/week) to keep enough events in each subgroup. Unhealthy waist circumference was defined as >102 cm in males or >88 cm in females [25]. Low grip strength was defined as lower than median in each gender (39 kg for males and 24 kg for females). Likelihood ratio tests were used to compare models with and without the interaction terms. Subgroup analysis was also conducted in participants of separate cancer site, considering that association between physical activity and mortality might vary in different organ systems [26].

Sensitivity analysis was conducted by excluding participants without a diagnosis of malignant neoplasms (codes C00-C97) and those with poor self-reported general health. Additional sensitivity analyses were performed by excluding participants with long-standing illness, disability or infirmity, and those died within two years since wearing accelerometer. Data were analyzed using R software Version 4.4.1 (R Development Core Team, Vienna, Austria). All statistical tests were two-sided, and statistical significance was defined as P <0.05.

## 3 Results

### 3.1 Baseline characteristics of study participants

The final analysis included 11,708 participants in UK Biobank, who had both cancer diagnosis records before wearing accelerometers and valid physical activity data (as shown in Supplementary Fig. S1). Malignance was definite in 10,123 participants (86.5%) and the top five primarily diagnosed malignance originated from skin, breast, genital system, digestive system and blood system (Supplementary Fig. S2A). Over a median follow-up of 8.9 (interquartile range 8.4–9.5) years, 983 (8.4%) death cases occurred, among which 656 (66.7%) were primarily attributed to cancer and 141 (14.3%) were related to cardiovascular diseases (shown in Supplementary Fig. S2B). Compared with those without cancer diagnosis, participants included in this study were older and had lower MVPA time and worse general health (Supplementary Table S2).

Table 1 presented the detailed baseline characteristics of all participants grouped by duration of MVPA. Participants with more MVPA time tended to be younger, have lower BMI, lower waist circumference and better general health condition. Other characteristics did not show distinctions of clinical significance. Baseline characteristics of participants grouped by duration of LPA and MPA exhibited a similar distribution trend (Supplementary Tables S3 and S4).

**Table 1:** Baseline characteristics of participants by quartiles of moderate to vigorous-intensity physical activity time.

	Quartiles of MVPA time (minutes/week)				P value
	<272	272–407	407–579	≥579	
*n*	2953	2905	2928	2922	
Age (years), mean (SD)	68.0 (6.1)	66.2 (6.8)	64.8 (7.0)	62.8 (7.4)	<0.001
Male, *n* (%)	1312 (44.4)	1233 (42.5)	1178 (40.2)	1062 (36.3)	<0.001
Townsend deprivation score, mean (SD)	−1.8 (2.8)	−2.0 (2.7)	−2.0 (2.7)	−1.9 (2.7)	0.14
Ethnicity, *n* (%)					0.067
White	2907 (98.4)	2854 (98.2)	2862 (97.7)	2849 (97.5)	
Asian	13 (0.4)	20 (0.7)	18 (0.6)	16 (0.5)	
Blacks	10 (0.3)	6 (0.2)	20 (0.7)	13 (0.4)	
Mixed	7 (0.2)	8 (0.3)	9 (0.3)	16 (0.5)	
Other	16 (0.5)	17 (0.6)	19 (0.6)	28 (1.0)	
Smoking status, *n* (%)					<0.001
Current	257 (8.7)	191 (6.6)	136 (4.6)	143 (4.9)	
Previous	1236 (41.9)	1134 (39.1)	1149 (39.2)	1113 (38.1)	
Never	1460 (49.4)	1580 (54.4)	1643 (56.1)	1666 (57.0)	
Alcohol intake frequency, *n* (%)					<0.001
Daily	712 (24.1)	709 (24.4)	724 (24.7)	674 (23.1)	
3–4 times/week	660 (22.3)	708 (24.4)	826 (28.2)	851 (29.1)	
1–2 times/week	688 (23.3)	714 (24.6)	723 (24.7)	744 (25.5)	
1–3 times/month	329 (11.2)	311 (10.7)	276 (9.4)	299 (10.2)	
Social drinking or never	564 (19.1)	463 (15.9)	379 (12.9)	354 (12.1)	
BMI (kg/m^2^), mean (SD)	28.2 (5.0)	27.0 (4.4)	26.2 (4.0)	25.2 (3.7)	<0.001
Waist circumference (cm), mean (SD)	93.2 (13.6)	89.5 (12.6)	87.0 (11.8)	83.5 (11.4)	<0.001
LPA (minute), mean (SD)	1622.6 (443.2)	1904.5 (404.7)	2037.9 (399.9)	2196.1 (401.4)	<0.001
MVPA (minute), mean (SD)	183.5 (63.6)	341.0 (38.1)	486.8 (49.2)	767.8 (174.7)	<0.001
Self-reported general health, *n* (%)					<0.001
Excellent	365 (12.4)	466 (16.0)	633 (21.6)	740 (25.3)	
Good	1716 (58.1)	1771 (61.0)	1818 (62.1)	1789 (61.2)	
Fair	712 (24.1)	580 (20.0)	422 (14.4)	363 (12.4)	
Poor	160 (5.5)	88 (3.0)	55 (1.9)	30 (1.0)	
Cancer duration (year), median (IQR)	7.1 (3.1, 12.7)	7.3 (3.1, 13.2)	7.0 (3.2, 12.7)	7.6 (3.4, 13.7)	0.014
Malignant, *n* (%)	2606 (88.2)	2537 (87.3)	2546 (87.0)	2436 (83.4)	<0.001
Other medical history, *n* (%)					
Cardiovascular diseases	1178 (39.9)	898 (30.9)	747 (25.5)	522 (17.9)	<0.001
Diabetes	229 (7.8)	96 (3.3)	48 (1.6)	53 (1.8)	<0.001
Long-standing illness	1328 (45.0)	1035 (35.6)	854 (29.2)	746 (25.5)	<0.001
Grip strength (kg), mean (SD)	29.5 (10.7)	30.2 (10.5)	30.4 (10.3)	30.3 (9.6)	0.002
FEV1, (liter), mean (SD)	2.6 (0.7)	2.7 (0.7)	2.8 (0.7)	2.8 (0.7)	<0.001

Data are expressed as mean (SD), median (IQR) or n (%), accordingly. BMI: body mass index; FEV1: forced expiratory volume in 1 second; LPA: light-intensity physical activity; MVPA: moderate to vigorous-intensity physical activity; SD: standard deviation.

### 3.2 Associations between duration and intensity of physical activity and mortality

Distribution of physical activity of different intensity was shown in Supplementary Fig. S3. There was a significant correlation between LPA, MPA and VPA time of individuals (Supplementary Fig. S4A–C), as were LPA and MVPA levels (Supplementary Fig. S4D).

**Fig. 1:**
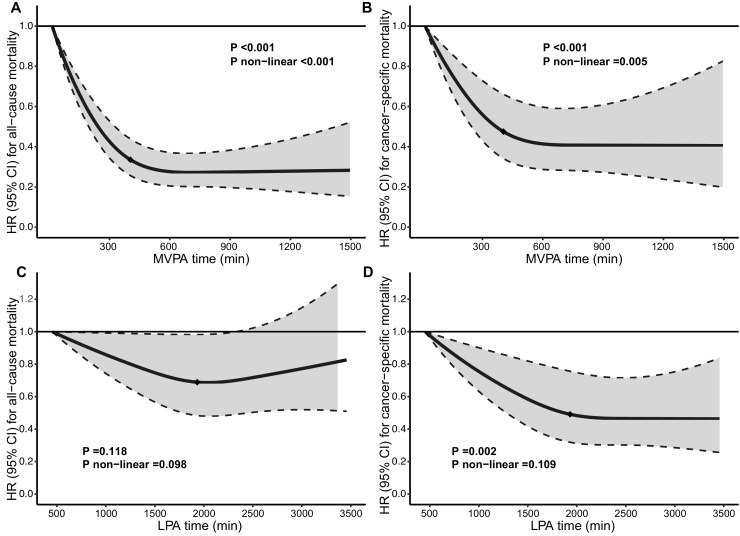
Dose-response associations between physical activity time and mortality among pan-cancer survivors. A & B: Dose-response associations between MVPA time and all-cause mortality and cancer-specific mortality, after adjusting for LPA time being adjusted. C & D: Dose-response associations between LPA time and all-cause mortality and cancer-specific mortality, after adjusting for MVPA time. All models additionally adjusted for age, sex, Townsend deprivation score, ethnicity, smoking status, alcohol intake frequency, BMI, waist circumference, self-reported general health, cancer duration, cardiovascular disease history, diabetes history, long-standing illness, disability or infirmity, grip strength, FEV1, and wear season. Bold lines represent HR and shaded areas between dashed lines indicate 95% CIs. Diamonds represent median values.

Significant non-linear dose-response associations of MVPA time with all-cause mortality (*P* < 0.001, *P*_non-linear_ <0.001) and cancer-specific mortality (*P* < 0.001, *P*_non-linear_ = 0.005) were discovered in restricted cubic splines (Fig. 1A–B). Comparing to participants with minimum MVPA time, risk of both all-cause mortality and cancer-specific mortality was reduced by more than 50% in those who reached median amount of MVPA time. Regarding LPA, no significant association was observed between LPA time and all-cause mortality (*P* = 0.118, *P*_non-linear_ = 0.098) while dose-response association between LPA time and cancer-specific mortality was significant (*P* = 0.002, *P*_non-linear_ <0.109) after adjusting MVPA time (Fig. 1C–D).

**Fig. 2:**
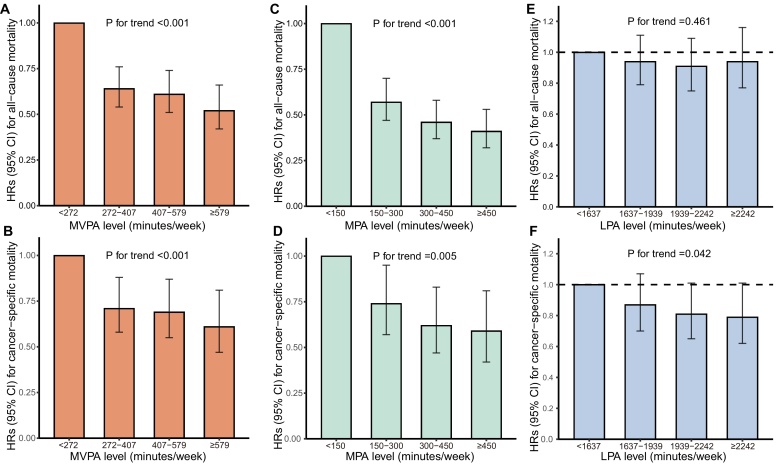
HRs and 95% CIs for mortality in participant groups divided by different levels of physical activity. A & B: associations between MVPA levels and all-cause mortality and cancer-specific mortality, after adjusting for LPA time (continuous variable). C & D: associations between MPA levels and all-cause mortality and cancer-specific mortality, after adjusting for LPA time (continuous variable) and VPA time (continuous variable). E & F: associations between LPA level and all-cause mortality and cancer-specific mortality, after adjusting for MVPA time (continuous variable). All models were also adjusted for age, sex, Townsend deprivation score, ethnicity, smoking status, alcohol intake frequency, BMI, waist circumference, self-reported general health, cancer duration, cancer biological behavior, cardiovascular disease history, diabetes history, long-standing illness, disability or infirmity, grip strength, FEV1 and wear season.

The cumulative incidence for mortality in groups categorized by different levels of physical activity was showed in Supplementary Fig. S5. By dividing all participants into four groups by quartiles of MVPA time, participants with more MVPA time had significantly lower risk of all-cause and cancer-specific mortality (P for trend <0.001, Fig. 2A and B). Specifically, 272–407, 407–579 and ≥579 min/week was associated with HRs of 0.64 (95% CI, 0.54–0.76), 0.61 (95% CI, 0.51–0.74) and 0.52 (95% CI, 0.42–0.66) for all-cause mortality and HRs of 0.71 (95% CI, 0.58–0.88), 0.69 (95% CI, 0.55–0.87) and 0.61 (95% CI, 0.47–0.81) for cancer-specific mortality, compared with <272 min/week (Supplementary Table S5). Dividing all participants into four groups by MPA time based on guideline recommendations,^23^ increased physical activity duration similarly demonstrated protective effect in both all-cause and cancer-specific death (Fig. 2C and D, Supplementary Table S5). However, no significantly reduced mortality was observed with increase of LPA time (Fig. 2E and F, Supplementary Table S5), although there was a decreasing tendency in cancer-specific mortality (P for trend = 0.042, Fig. 2F).

### 3.3 Subgroup analyses

Associations between MVPA time (≥272 vs <272 min/week) and mortality were similar in age, gender, BMI, waist circumference, grip strength, FEV1 and cancer duration subgroups, indicating no significant interactions (Supplementary Fig. S6 and Tables S6 and S7). Participants with non-malignant diagnoses benefited more in terms of all-cause death than those with malignant diagnoses (P for interaction = 0.021), while confidence interval crossed the invalid value. Associations between LPA time (≥1637 vs <1637 min/week) and mortality were similar in all subgroups and there was a tendency that females, and participants with BMI lower than 30 kg/m^2^ and with cancer duration lower than 5 years could benefit from LPA (Supplementary Tables S8 and S9).

**Fig. 3:**
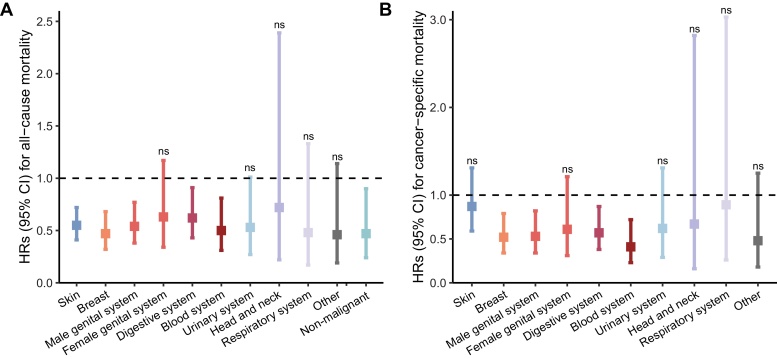
HRs and 95% CI for mortality in participants with cancer diagnoses of different sites. A: association between MVPA time (≥272 vs <272 min/week) and all-cause mortality. B: association between MVPA time (≥272 vs <272 min/week) and cancer-specific mortality. All HRs and 95% CIs were calculated in multivariate Cox proportional hazards models that adjusted for LPA time (continuous variable), age, sex and BMI.

Regarding different cancer sites, increased MVPA time was associated with reduced all-cause mortality of cancers originating from skin, breast, male genital system, digestive system, blood system as well as cancers with non-malignant behaviors, ranging from 38% to 53% (Fig. 3A and Supplementary Table S10). There was a similar tendency for cancers originating from urinary system but without statistical significance. Meanwhile, cancer-specific mortality of cancers originating from breast, male genital system, digestive system and blood system in participants with MVPA time ≥272 min were reduced by 43% to 59% (Fig. 3B and Supplementary Table S11). Increased LPA time was not associated with significantly reduced all-cause mortality and cancer-specific mortality in all cancer types (Supplementary Tables S12 and S13).

### 3.4 Sensitivity analyses

Summary of participants in sensitivity analyses were presented in Supplementary Tables S14–S17. Associations between MVPA time and MPA time with mortality were consistent with main analyses after excluding participants with non-malignant diagnosis, participants with poor general health and long-standing illness, and those died within two years since wearing accelerometer (Supplementary Tables S18–S20). Moreover, there was no significant survival benefit with the increase of LPA time (Supplementary Tables S18–S20).

## 4 Discussion

In this large prospective cohort study, we comprehensively investigated the associations between postdiagnosis accelerometer-derived physical activity and mortality among cancer survivors. Following rigorous multivariable adjustment, our analysis revealed that cancer survivors achieving adequate MPA or MVPA demonstrated significantly reduced risks of both all-cause and cancer-specific mortality. In contrast, LPA showed only marginal protective effects, even at the highest observed physical activity levels. This is, to our knowledge, the largest study to explore the dose-response associations between physical activity time and survival among cancer survivors. The conclusions reinforced the distinct benefits of physical activity, and benefits were significant only when the intensity reached certain levels [27].

Piles of evidence has demonstrated that physical activity is impressively beneficial in general population [8,[28], [29], [30]]. Recently, several researches concentrating on cancer population consistently reported that active physical activity, as an important part of healthy lifestyle, was associated with reduced all-cause and cancer-specific mortality [[12], [13], [14],^27^]. For example, Lavery et al. leveraged data from the Prostate, Lung, Colorectal, and Ovarian (PLCO) cancer screening trial and found that exercise consistent with guidelines yielded a 25% reduced risk of all-cause mortaity and a 21% reduced risk of cancer-specific mortality [14]. However, in these studies, physical activity data self-reported, which might influence the precision of exposure evaluation and limited the extension of dose-response analyses. The World Health Organization Guidelines Development Group has proposed the need for investigations using wearable devices to objectively assess the relationship between physical activity and mortality [31]. All participants in this study had valid device-derived physical activity data, and the analysis revealed that increased MPVA and MPA time correlated with significant benefits. Additionally, cancer survivors with low grip strength or FEV1 benefited to the same extent compared to those with high measurements (Supplementary Fig. S6). Grip strength and FEV1 were important indicators of muscle strength and lung function, respectively, so our results indicated physical activity was universally beneficial even in cancer survivors who had lower muscle strength and poorer cardiopulmonary fitness [[32], [33], [34], [35]].

Further analysis revealed that LPA alone was not associated with survival after adjusting MVPA time. Although a significant and linear association was detected between LPA time and cancer-specific mortality in restricted cubic splines (Fig. 1D), the effect turned non-significant in subsequent sensitivity analyses. A similar trend was reported in a previous study [36]. A study which recruited cancer survivors from the National Health and Nutrition Examination Survey (NHANES) concluded that lighter-intensity activities were beneficial, whereas only those with LPA > 2.3 h/day had benefits compared to LPA < 1.5 h/day and half of the participants with increased LPA time did not have significant survival benefit [15]. LPA appears to be favorably associated with improvement in cardiovascular fitness (CRF) and lower BMI, while previous study reported non-significant associations between LPA time and high-density lipoprotein (HDL) and triglycerides, suggesting that the metabolic benefits of LPA were delicate [37,38]. In subgroup analyses, we found that females, participants with lower BMI and with shorter cancer duration would benefit from LPA, supported by the sex-specific difference that women benefited more from physical activity [39].

Analysis across different cancer sites disclosed variability in cancer response to physical activity. Obvious survival benefits were observed in cancers from breast, male genital system, digestive system and hematopoietic system for both all-cause and cancer-specific mortality. All-cause mortality of cancers from skin was improved but not cancer-specific mortality. Similarly, Cao et al. also concluded that there was no association between physical activity and cancer-specific mortality despite of significant association for all-cause mortality in skin cancer cancers from United States [40]. Exercise might reduce the cancer-specific mortality by boosting immune system, restraining tumor metastasis and modulating hormone secretion [[41], [42], [43]]. Heterogeneity in the immune microenvironment and peripheral autonomic nerves in different cancer types might partially account for variability in cancer response [44,45]. We also found non-significant associations between active physical activity and survival in cancers from urinary, head and neck as well as respiratory system, while this conclusion must be interpreted with caution, given the small sample of these cancer types. Further investigations focusing on these systems are warranted considering the lack of current evidence [5].

This study has several strengths in this study. Intensity and duration of postdiagnosis physical activity was objectively measured by wearable devices and all data was valid, minimizing the recall and report bias. Large sample size and sporadic loss to follow-up made it feasible for covariate adjustment to control potential confounders [46]. Moreover, credible registers of cancer diagnosis as well as primary causes of death enabled subgroup analysis across individual cancer sites to investigate the variability in response to physical activity. However, several limitations of this study should be noted. Firstly, most cancer diagnoses originated from skin, breast, genital system and digestive system, while cases from respiratory system, head and neck and central nervous system were limited. Therefore, further investigations are advocated to explore associations between physical activity and these cancer types. Secondly, participants of this study were predominantly Europeans, hence, generalization to other districts like Asia and Africa might be limited. Thirdly, most cancer survivors in this study had relatively longer survival following the initial diagnosis, reflecting generally good prognosis and easily available medical resources [47]. However, subgroup analysis conducted in participants with cancer duration less than 5 years showed that this group benefited as much from active physical activity as those with longer survival, indicating that both newly and previously diagnosed cancer patients benefited from active physical activity. Next, the data of physical activity was collected over a single period of 7 day at baseline, which may not fully capture changes of physical activity over time. Nevertheless, previous researches demonstrated high consistency in repeated physical activity measurements over several months to 4 years [48]. Validity of the conclusions might not be impacted by this limitation substantially. Last but not least, previous cancer treatments, which might influence cancer-specific mortality of cancer survivors, were not adjusted in the study, necessitating cautious interpretation of these findings.

## 5 Conclusions

In summary, findings from this study verified the inverse associations between objectively measured physical activity time after diagnosis and mortality in cancer survivors and highlighted the important role of active physical activity on long-term management of cancer, but survival benefits were of clinical significance only when physical activity reached certain intensity.

## Ethics approval and consent to participate

Informed consent was obtained from all participants in the UK Biobank, and the study received ethical approvals from the NHS National Research Ethics Service (Ref 11/NW/0382). The current secondary analysis was approved by the UK Biobank (Application ID 107479). Participants gave informed consent to participate in the study before taking part.

## Funding

This work was supported by the 10.13039/501100001809National Natural Science Foundation of China (No. 62473005).

## Availability of data and materials

The main data used in this study were accessed from the publicly available UK Biobank Resource under application number 107479. The UK Biobank data can be accessed by researchers on the application.

## CRediT authorship contribution statement

**Zhihan Jiang:** Writing - original draft, Validation, Formal analysis, Conceptualization. **Bingyan Wang:** Visualization, Validation. **Yifei Zhao:** Supervision, Data curation. **Jing Weng:** Funding acquisition, Data curation. **Jiaojiao Liao:** Software, Methodology, Data curation. **Liyuan Tao:** Supervision, Data curation. **Kui Sun:** Visualization. **Zhipeng Zhang:** Writing - review & editing, Project administration, Funding acquisition. **Xin Zhou:** Writing - review & editing, Funding acquisition. **Wei Fu:** Supervision, Project administration, Funding acquisition, Data curation, Conceptualization.

## Declaration of competing interest

The authors declare that they have no known competing financial interests or personal relationships that could have appeared to influence the work reported in this paper.
