# Supplementary material for: Association between accelerometer-measured physical activity and mortality in cancer survivors: A prospective cohort study from UK Biobank
Source: J Nutr Health Aging. 2025 May 26;29(8):100586. doi: 10.1016/j.jnha.2025.100586 (PMC12172998; doi:10.1016/j.jnha.2025.100586)
Supplement: Supplementary file 1 [file mmc1.docx]

**Association between accelerometer-measured physical activity and mortality in cancer survivors: a prospective cohort study from UK Biobank**

**Supplementary Contents**

**Figures**

[Supplementary Methods 3](#_Toc197475902)

[Figure S1. Flowchart of participant sample. 5](#_Toc197475903)

[Figure S2. Distribution of primarily diagnosed cancer types in baseline and death causes. 6](#_Toc197475904)

[Figure S3. Distribution of physical activity of different intensity. 7](#_Toc197475905)

[Figure S4. Correlation between physical activity time of different intensity. 8](#_Toc197475906)

[Figure S5. Cumulative incidence for all-cause and cancer-specific mortality divided by different levels of physical activity. 9](#_Toc197475907)

[Figure S6. Subgroup analyses between MVPA time (≥272 vs <272 minutes/week) and mortality. 10](#_Toc197475908)

**Tables**

[Table S1. All diagnosed cancer types and according ICD-10 codes. 11](#_Toc197475909)

[Table S2. Baseline characteristics of enrolled participants compared to those without cancer diagnosis. 12](#_Toc197475910)

[Table S3. Baseline characteristics of participants by quartiles of LPA time. 13](#_Toc197475911)

[Table S4. Baseline characteristics of participants grouped by of recommended MPA time. 14](#_Toc197475912)

[Table S5. HRs and 95% CIs for mortality in participant groups divided by different levels of physical activity. 15](#_Toc197475913)

[Table S6. Subgroup analysis between MVPA time (≥272 vs <272 minutes/week) and all-cause mortality. 16](#_Toc197475914)

[Table S7. Subgroup analysis between MVPA time (≥272 vs <272 minutes/week) and cancer-specific mortality. 17](#_Toc197475915)

[Table S8. Subgroup analysis between LPA time (≥1637 vs <1637 minutes/week) and all-cause mortality. 18](#_Toc197475916)

[Table S9. Subgroup analysis between LPA time (≥1637 vs <1637 minutes/week) and cancer-specific mortality. 19](#_Toc197475917)

[Table S10. Subgroup analysis between MVPA time (≥272 vs <272 minutes/week) and all-cause mortality in different sites. 20](#_Toc197475918)

[Table S11. Subgroup analysis between MVPA time (≥272 vs <272 minutes/week) and cancer-specific mortality in different sites. 21](#_Toc197475919)

[Table S12. Subgroup analysis between LPA time (≥1637 vs <1637 minutes/week) and all-cause mortality in different sites. 22](#_Toc197475920)

[Table S13. Subgroup analysis between LPA time (≥1637 vs <1637 minutes/week) and cancer-specific mortality in different sites. 23](#_Toc197475921)

[Table S14. Methods of sensitivity analysis and outcome event summary. 24](#_Toc197475922)

[Table S15. Baseline characteristics of participants by quartiles of MVPA time after excluding non-malignance and those with poor self-reported medical condition (n=9823). 25](#_Toc197475923)

[Table S16. Baseline characteristics of participants by quartiles of MVPA time after excluding non-malignance, those with poor self-reported medical condition and long-standing illness (n=6595). 26](#_Toc197475924)

[Table S17. Baseline characteristics of participants by quartiles of MVPA time after excluding non-malignance, those with poor self-reported medical condition, with long-standing illness, and died within two years since wearing accelerometer (n=6503). 27](#_Toc197475925)

[Table S18. Cox regression results of sensitivity analysis that excluded non-malignant participants and those with poor self-reported medical condition. 28](#_Toc197475926)

[Table S19. Cox regression results of sensitivity analysis that excluded non-malignant participants, those with poor self-reported medical condition and long-standing illness. 29](#_Toc197475927)

[Table S20. Cox regression results of sensitivity analysis that excluded non-malignant participants, those with poor self-reported medical condition, with long-standing illness, and died within two years since wearing accelerometer. 30](#_Toc197475928)

# Supplementary Methods

**Inclusion criteria:**

**1. Definite cancer diagnosis**

Cancer data linkage was obtained through national cancer registries. In "Cancer register" domain of UK Biobank, cancer diagnosis coded by ICD-10 (field ID 40006) and date of cancer diagnosis (field ID 40005) were utilized to determine cancer diagnosis for each participant. Code of cancer diagnosis in the present study contained C00-C97, D00-D09, D10-D36, and D37-D48.

**2. Valid accelerometer-measured physical activity data**

Participants who had valid accelerometer data were included, and valid accelerometer data were defined as meeting all condition below: A. total wear time ≥72 hours with wear data recorded in each one-hour period of the 24-hour cycle (field ID 90015); B. raw accelerometer data being well calibrated (field ID 90016); C. sufficient data to conduct calibration on the participant's own stationary data (none of the three sensor axes exceeding a +/- 300 milli-gravity range, field ID 90017).

**Exclusion criteria:**

**1. No cancer diagnosis when wearing accelerometer**

Participants whose cancer diagnosis occurred later than wearing accelerometer were excluded. For participants diagnosed with cancer at multi sites on different dates, first cancer diagnosis date was counted.

**2. Missing covariate data**

Participants with missing covariate data were excluded instead of imputation. Covariates with missing values included FEV1 derived from spirometry test (field ID 3063), grip strength (field ID 46 and 47), BMI (field ID 21001), self-reported medical condition (field ID 2178), and waist circumference (field ID 48).

**Accelerometer-measured physical activity**

Participants were asked to wear an Axivity AX3 triaxial accelerometer on their dominant wrist for 7 consecutive days at all times. Then, accelerometers were sending back to coordinating center of UK Biobank for extracting and calibrating the raw data. The sensor captured acceleration for 7 days at a frequency of 100 Hz with a dynamic range of ±8 g, and the signals were calibrated to gravity.^1^ The average vector magnitude was processed by combining sampling data into 5 s epochs, and overall mean acceleration represents the global PA.^2^ Non-wear time was considered to be time periods of ≥60 min where the standard deviation of acceleration in each of the three axes was <13 milligravities (mg).^2^ Missing data due to non-wear time were imputed on the basis of similar time-of-day segments from that individual. Minutes per week of light-intensity physical activity, moderate-intensity physical activity, and vigorous-intensity physical activity were determines as time spend in 30–125 mg, >125–400 mg, and >400 mg intensity activity, respectively.^3,4^

**Reference**

1. Doherty A, Jackson D, Hammerla N, et al. Large Scale Population Assessment of Physical Activity Using Wrist Worn Accelerometers: The UK Biobank Study. PLoS One 2017; 12(2): e0169649.

2. Ahn HJ, Choi EK, Rhee TM, et al. Accelerometer-derived physical activity and the risk of death, heart failure, and stroke in patients with atrial fibrillation: a prospective study from UK Biobank. Br J Sports Med 2024; 58(8): 427-34.

3. Ho FK, Zhou Z, Petermann-Rocha F, et al. Association Between Device-Measured Physical Activity and Incident Heart Failure: A Prospective Cohort Study of 94 739 UK Biobank Participants. Circulation 2022; 146(12): 883-91.

4. Strain T, Wijndaele K, Dempsey PC, et al. Wearable-device-measured physical activity and future health risk. Nat Med 2020; 26(9): 1385-91.

# Figure S1. Flowchart of participant sample.

^^

Among all participants (N=502,236) from UK Biobank, a subset of population with cancer diagnosis (ICD-10: C00-C97, D00-D09, D10-D36, and D37-D48) was included preliminarily (N=119,326). Cancer data linkage was obtained through national cancer registries. Then participants without accelerometer-measured data (N=96,157) and those without enough wear time (N=1,455) were further excluded. Next, participants whose first cancer diagnosis occurred later than wearing accelerometer (N=8,710) were excluded. Eventually, after excluding 1,296 participants with missing covariate data, 11,708 cancer survivors were included in final analysis. Additionally, 69,197 participants with accelerometer-measured data but without cancer diagnosis during follow-up were picked out, as indicated in dashed box. Baseline characteristics of these participants were compared with that of participants included in the study.

^a^ Invalid accelerometer data is defined as total wear time <72 hours, no wear data in each one-hour period of the 24-hour cycle, or poor calibration.

^b^ Participants in UK Biobank may have more than one cancer diagnoses and only participants whose first diagnoses of cancer were later than their accelerometer-wearing date were excluded.

# Figure S2. Distribution of primarily diagnosed cancer types in baseline and death causes.


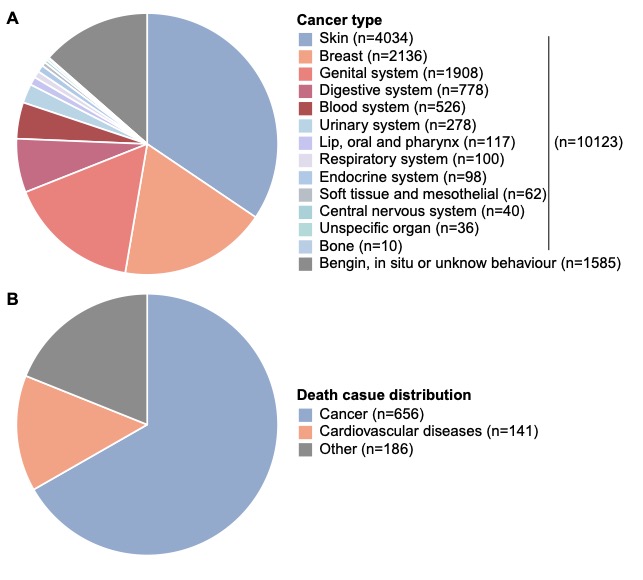


One participant may have cancer diagnosis of multiple sites. Only first cancer diagnosis was summarized here. All cancer diagnosis was summarized in Table S1.

# Figure S3. Distribution of physical activity of different intensity.

**
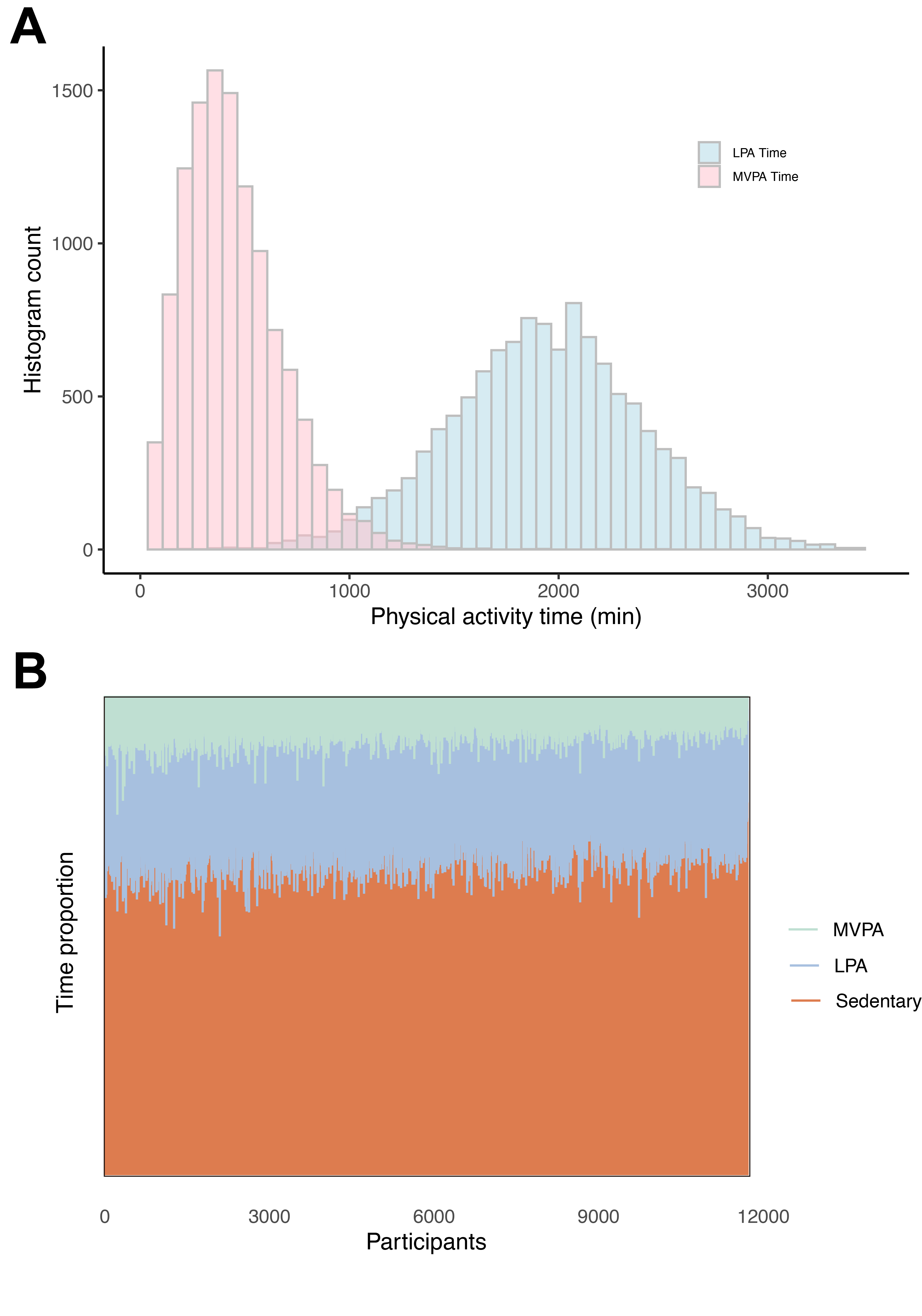
**

A, histogram showed the distribution of LPA time (blue) and MVPA time (red), respectively. B, stack diagram delineated the proportion of sedentary time (orange), LPA time (blue) and MVPA time (green) for individuals.

# Figure S4. Correlation between physical activity time of different intensity.





A-C, correlation between LPA, MPA and VPA, and the correlation coefficients were calculated by Pearson correlation. D, correlation between LPA and MPA levels, indicating a significant correlation between LPA time and MVPA time.

# Figure S5. Cumulative incidence for all-cause and cancer-specific mortality divided by different levels of physical activity.

**
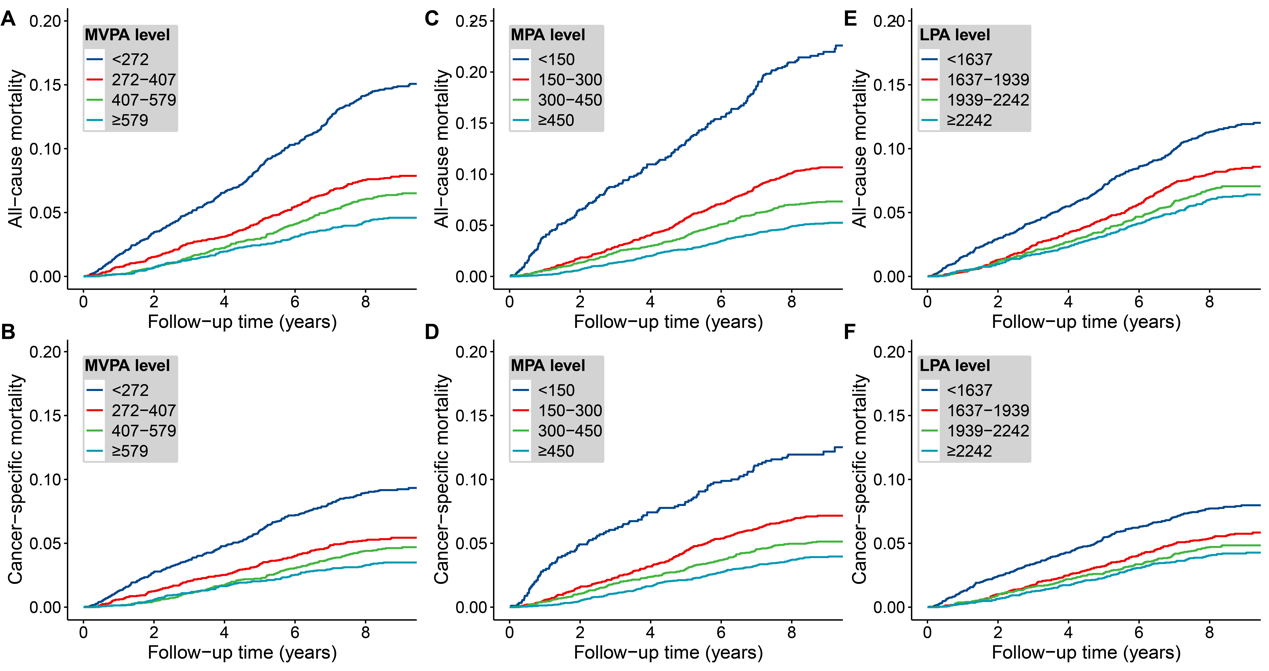
**

Y-axis showed incidence of mortality as a percentage of all participants at a certain follow-up time and only part of Y-axis was exhibited.

# Figure S6. Subgroup analyses between MVPA time (≥272 vs <272 minutes/week) and mortality.


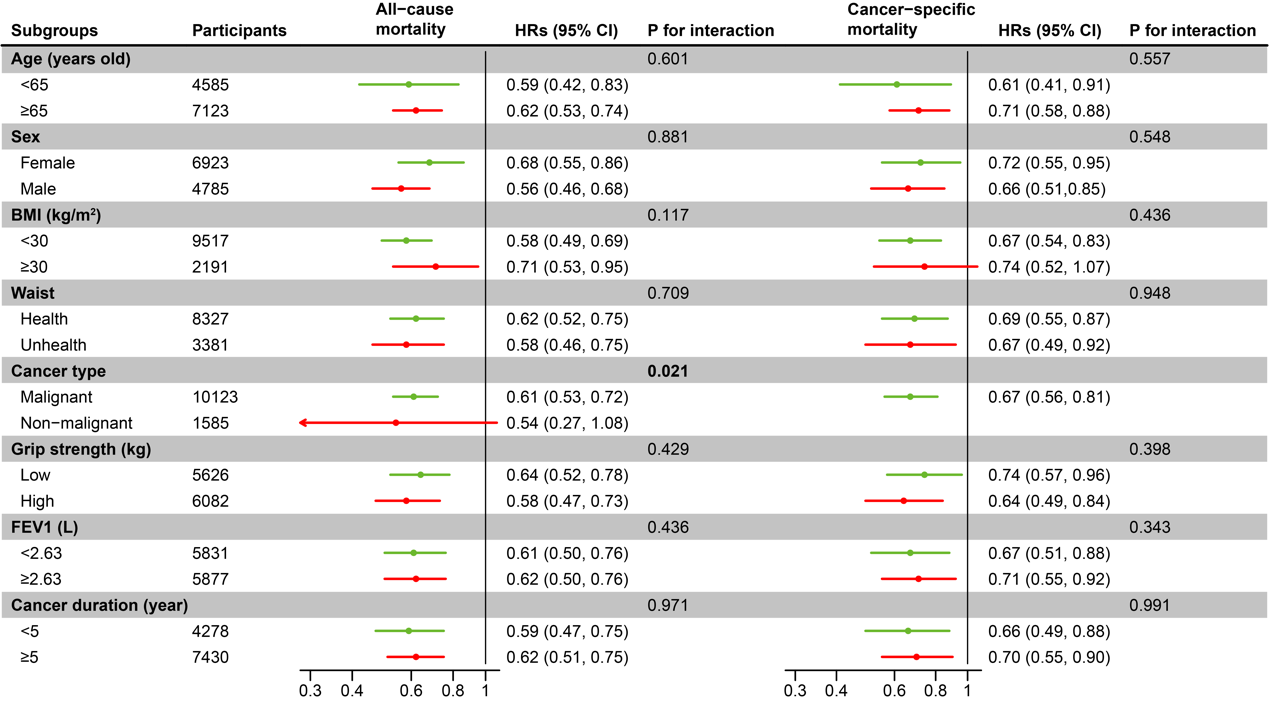


All HRs and 95% CIs were calculated in multivariate Cox proportional hazards models that adjusted for LPA time (continuous variable), age, sex, Townsend deprivation score, ethnicity, smoking status, alcohol intake frequency, BMI, waist circumference, self-reported general health, cancer duration, cancer biological behavior, cardiovascular disease history, diabetes history, long-standing illness, disability or infirmity, grip strength, FEV1 and wear season. HR for cancer-specific mortality in non-malignant was not calculated due to the small number of events. P values for interaction were calculated by likelihood ratio tests.

# Table S1. All diagnosed cancer types and according ICD-10 codes.

| **Cancer types** | **Participants** | **ICD-10 codes** |
| --- | --- | --- |
| **Skin** | 4094 | C43-C44 |
| **Breast** | 2163 | C50 |
| **Genital system** | 1971 |  |
| Female genital | 505 | C51-C58 |
| Male genital^a^ | 1466 | C60-C63 |
| **Digestive system** | 794 | C15-C26 |
| **Blood system** | 539 | C81-C96 |
| **Urinary system** | 295 | C64-C68 |
| **Lip, oral and pharynx** | 122 | C00-C14 |
| **Respiratory system** | 101 | C30-C39 |
| **Endocrine system** | 99 | C73-C75 |
| **Soft tissue and mesothelial** | 67 | C45-C49 |
| **Central nervous system** | 40 | C69-C72 |
| **Unspecific organ** | 36 | C76-C80 |
| **Bone** | 12 | C40-C41 |
| **Benign, in situ or unknow behaviour** | 1585 | D00-D48 |

Sum of counts was larger than number of participants (n = 11708) for multiple cancer sites in each individual. Only diagnoses before accelerometer-wearing date were counted in this study.

^a^Cancers originating from male genital system were mainly prostate cancer (C61, n = 1375)

# Table S2. Baseline characteristics of enrolled participants compared to those without cancer diagnosis.

|  | **Participants with accelerometer-measured physical activity** | |  | **P value** |
| --- | --- | --- | --- | --- |
|  | **Without cancer** | **With cancer** | |  |
| n | 69197 | 11708 | |  |
| **Age (years), mean (SD)** | 61.4 (7.9) | 65.5 (7.1) | | <0.001 |
| **Male, n (%)** | 29601 (42.8) | 4785 (40.9) | | <0.001 |
| **Townsend deprivation score, mean (SD)** | -1.7 (2.8) | -1.9 (2.7) | | <0.001 |
| **Ethnicity, n (%)** |  |  | | <0.001 |
| White | 66583 (96.2) | 11472 (98.0) | |  |
| Asian | 932 (1.3) | 67 (0.6) | |  |
| Blacks | 650 (0.9) | 49 (0.4) | |  |
| Mixed | 427 (0.6) | 40 (0.3) | |  |
| Other | 602 (0.9) | 80 (0.7) | |  |
| **Smoking status, n (%)** |  |  | | <0.001 |
| Current | 4757 (6.9) | 727 (6.2) | |  |
| Previous | 23862 (34.5) | 4632 (39.6) | |  |
| Never | 40578 (58.6) | 6349 (54.2) | |  |
| **Alcohol intake frequency, n (%)** |  |  | | <0.001 |
| Daily | 15331 (22.2) | 2819 (24.1) | |  |
| 3-4 times/week | 18110 (26.2) | 3045 (26.0) | |  |
| 1-2 times/week | 17570 (25.4) | 2869 (24.5) | |  |
| 1-3 times/month | 7655 (11.1) | 1215 (10.4) | |  |
| Social drinking or never | 10531 (15.2) | 1760 (15.0) | |  |
| **BMI (kg/m^2^), mean (SD)** | 26.6 (4.5) | 26.6 (4.5) | | 0.910 |
| **Waist circumference (cm), mean (SD)** | 87.9 (13.0) | 88.3 (12.9) | | 0.001 |
| **LPA (minute), mean (SD)** | 1962.1 (462.1) | 1939.5 (463.5) | | <0.001 |
| **MVPA (minute), mean (SD)** | 502.4 (253.2) | 444.2 (236.6) | | <0.001 |
| **Self-reported general health, n (%)** |  |  | |  |
| Excellent | 15892 (23.0) | 2204 (18.8) | | <0.001 |
| Good | 41511 (60.0) | 7094 (60.6) | |  |
| Fair | 10260 (14.8) | 2077 (17.7) | |  |
| Poor | 1524 (2.2) | 333 (2.8) | |  |
| **Other medical history, n (%)** |  |  | |  |
| Cardiovascular diseases | 15936 (23.0) | 3345 (28.6) | | <0.001 |
| Diabetes | 2211 (3.2) | 426 (3.6) | | 0.014 |
| Long-standing illness | 17768 (25.7) | 3963 (33.8) | | <0.001 |
| **Grip strength (kg), mean (SD)** | 31.31 (10.74) | 30.08 (10.32) | | <0.001 |
| **FEV1, (liter), mean (SD)** | 2.83 (0.76) | 2.70 (0.72) | | <0.001 |

BMI, body mass index; FEV1, forced expiratory volume in 1 second; IQR, interquartile range; LPA, light-intensity physical activity; MVPA moderate to vigorous-intensity physical activity; SD standard deviation.

# Table S3. Baseline characteristics of participants by quartiles of LPA time.

|  | Quartiles of LPA time (minutes/week) | | | | P value |
| --- | --- | --- | --- | --- | --- |
|  | <1637 | 1637-1939 | 1939-2242 | ≥2242 |  |
| n | 2939 | 2921 | 2932 | 2916 |  |
| **Age (years), mean (SD)** | 65.9 (7.1) | 65.5 (7.2) | 65.2 (7.1) | 65.3 (6.9) | 0.001 |
| **Male, n (%)** | 1552 (52.8) | 1300 (44.5) | 1093 (37.3) | 840 (28.8) | <0.001 |
| **Townsend deprivation score, mean (SD)** | -1.7 (2.9) | -1.9 (2.7) | -2.0 (2.6) | -2.1 (2.6) | <0.001 |
| **Ethnicity, n (%)** |  |  |  |  | 0.41 |
| White | 2874 (97.8) | 2875 (98.4) | 2871 (97.9) | 2852 (97.8) |  |
| Asian | 20 (0.7) | 16 (0.5) | 15 (0.5) | 16 (0.5) |  |
| Blacks | 11 (0.4) | 8 (0.3) | 11 (0.4) | 19 (0.7) |  |
| Mixed | 9 (0.3) | 7 (0.2) | 11 (0.4) | 13 (0.4) |  |
| Other | 25 (0.9) | 15 (0.5) | 24 (0.8) | 16 (0.5) |  |
| **Smoking status, n (%)** |  |  |  |  | <0.001 |
| Current | 241 (8.2) | 175 (6.0) | 160 (5.5) | 151 (5.2) |  |
| Previous | 1188 (40.4) | 1197 (41.0) | 1126 (38.4) | 1121 (38.4) |  |
| Never | 1510 (51.4) | 1549 (53.0) | 1646 (56.1) | 1644 (56.4) |  |
| **Alcohol intake frequency, n (%)** |  |  |  |  | 0.001 |
| Daily | 709 (24.1) | 663 (22.7) | 725 (24.7) | 722 (24.8) |  |
| 3-4 times/week | 702 (23.9) | 786 (26.9) | 799 (27.3) | 758 (26.0) |  |
| 1-2 times/week | 703 (23.9) | 760 (26.0) | 715 (24.4) | 691 (23.7) |  |
| 1-3 times/month | 326 (11.1) | 313 (10.7) | 265 (9.0) | 311 (10.7) |  |
| Social drinking or never | 499 (17.0) | 399 (13.7) | 428 (14.6) | 434 (14.9) |  |
| **BMI (kg/m^2^), mean (SD)** | 27.67 (4.76) | 26.83 (4.49) | 26.27 (4.12) | 25.77 (4.2) | <0.001 |
| **Waist circumference (cm), mean (SD)** | 92.3 (13.2) | 88.9 (12.7) | 87.0 (12.3) | 85.0 (12.2) | <0.001 |
| **LPA (minute), mean (SD)** | 1352.3 (238.7) | 1795.4 (86.4) | 2088.7 (84.9) | 2525.7 (236.6) | <0.001 |
| **MVPA (minute), mean (SD)** | 296.8 (181.1) | 421.6 (204.4) | 490.6 (229.9) | 569.1 (237.8) | <0.001 |
| **Self-reported general health, n (%)** | 463 (15.8) | 585 (20.0) | 570 (19.4) | 586 (20.1) | <0.001 |
| Excellent | 1682 (57.2) | 1765 (60.4) | 1821 (62.1) | 1826 (62.6) |  |
| Good | 645 (21.9) | 502 (17.2) | 484 (16.5) | 446 (15.3) |  |
| Fair | 149 (5.1) | 69 (2.4) | 57 (1.9) | 58 (2.0) |  |
| Poor | 27.7 (4.8) | 26.8 (4.5) | 26.3 (4.1) | 25.8 (4.2) | <0.001 |
| **Cancer duration (year), median (IQR)** | 7.0 (2.8, 12.3) | 6.9 (3.1, 12.7) | 7.5 (3.4, 13.6) | 7.6 (3.50, 14.0) | <0.001 |
| **Malignant, n (%)** | 2579 (87.8) | 2560 (87.6) | 2506 (85.5) | 2478 (85.0) | 0.001 |
| **Other medical history, n (%)** |  |  |  |  |  |
| Cardiovascular diseases | 1024 (34.8) | 840 (28.8) | 783 (26.7) | 698 (23.9) | <0.001 |
| Diabetes | 170 (5.8) | 114 (3.9) | 72 (2.5) | 70 (2.4) | <0.001 |
| Long-standing illness | 1201 (40.9) | 966 (33.1) | 926 (31.6) | 870 (29.8) | <0.001 |
| **Grip strength (kg), mean (SD)** | 32.0 (10.9) | 30.6 (10.3) | 29.8 (10.1) | 27.9 (9.5) | <0.001 |
| **FEV1, (liter), mean (SD)** | 2.8 (0.7) | 2.8 (0.7) | 2.7 (0.7) | 2.6 (0.7) | <0.001 |

BMI, body mass index; FEV1, forced expiratory volume in 1 second; IQR, interquartile range; LPA, light-intensity physical activity; MVPA moderate to vigorous-intensity physical activity; SD standard deviation.

# Table S4. Baseline characteristics of participants grouped by of recommended MPA time.

|  | Quartiles of MPA time (minutes/week) | | | | P value |
| --- | --- | --- | --- | --- | --- |
|  | <150 | 150-300 | 300-450 | ≥450 |  |
| n | 903 | 2905 | 3353 | 4547 |  |
| **Age (years), mean (SD)** | 68.9 (5.8) | 67.2 (6.4) | 65.6 (7.0) | 63.5 (7.3) | <0.001 |
| **Male, n (%)** | 427 (47.3) | 1267 (43.6) | 1428 (42.6) | 1663 (36.6) | <0.001 |
| **Townsend deprivation score, mean (SD)** | -1.6 (2.9) | -1.9 (2.7) | -2.0 (2.7) | -1.9 (2.7) | <0.001 |
| **Ethnicity, n (%)** |  |  |  |  | 0.148 |
| White | 892 (98.8) | 2859 (98.4) | 3284 (97.9) | 4437 (97.6) |  |
| Asian | 2 (0.2) | 14 (0.5) | 26 (0.8) | 25 (0.5) |  |
| Blacks | 2 (0.2) | 10 (0.3) | 11 (0.3) | 26 (0.6) |  |
| Mixed | 2 (0.2) | 8 (0.3) | 8 (0.2) | 22 (0.5) |  |
| Other | 5 (0.6) | 14 (0.5) | 24 (0.7) | 37 (0.8) |  |
| **Smoking status, n (%)** |  |  |  |  | <0.001 |
| Current | 104 (11.5) | 204 (7.0) | 190 (5.7) | 229 (5.0) |  |
| Previous | 393 (43.5) | 1179 (40.6) | 1299 (38.7) | 1761 (38.7) |  |
| Never | 406 (45.0) | 1522 (52.4) | 1864 (55.6) | 2557 (56.2) |  |
| **Alcohol intake frequency, n (%)** |  |  |  |  | <0.001 |
| Daily | 223 (24.7) | 683 (23.5) | 820 (24.5) | 1093 (24.0) |  |
| 3-4 times/week | 186 (20.6) | 669 (23.0) | 894 (26.7) | 1296 (28.5) |  |
| 1-2 times/week | 205 (22.7) | 702 (24.2) | 832 (24.8) | 1130 (24.9) |  |
| 1-3 times/month | 99 (11.0) | 323 (11.1) | 339 (10.1) | 454 (10.0) |  |
| Social drinking or never | 190 (21.0) | 528 (18.2) | 468 (14.0) | 574 (12.6) |  |
| **BMI (kg/m^2^), mean (SD)** | 29.2 (5.4) | 27.7 (4.7) | 26.6 (4.3) | 25.5 (3.8) | <0.001 |
| **Waist circumference (cm), mean (SD)** | 96.3 (14.4) | 91.4 (12.7) | 88.6 (12.5) | 84.6 (11.6) | <0.001 |
| **LPA (minute), mean (SD)** | 1381.2 (428.8) | 1754.5 (404.8) | 1959.4 (400.5) | 2153.9 (404.7) | <0.001 |
| **MPA (minute), mean (SD)** | 101.9 (36.2) | 231.5 (42.8) | 372.6 (42.3) | 638.2 (167.0) | <0.001 |
| **MVPA (minute), mean (SD)** | 104.4 (38.3) | 241.7 (47.7) | 392.8 (51.7) | 679.2 (184.7) | <0.001 |
| **Self-reported general health, n (%)** | 90 (10.0) | 398 (13.7) | 615 (18.3) | 1101 (24.2) | <0.001 |
| Excellent | 452 (50.1) | 1790 (61.6) | 2068 (61.7) | 2784 (61.2) |  |
| Good | 284 (31.5) | 604 (20.8) | 592 (17.7) | 597 (13.1) |  |
| Fair | 77 (8.5) | 113 (3.9) | 78 (2.3) | 65 (1.4) |  |
| Poor | 29.17 (5.38) | 27.66 (4.72) | 26.62 (4.26) | 25.5 (3.8) | <0.001 |
| **Cancer duration (year), median (IQR)** | 6.9 (2.9, 12.3) | 7.2 (3.2, 13.0) | 7.2 (3.2, 13.0) | 7.4 (3.3, 13.4) | 0.054 |
| **Malignant, n (%)** | 795 (88.0) | 2571 (88.5) | 2896 (86.4) | 3861 (84.9) | <0.001 |
| **Other medical history, n (%)** |  |  |  |  |  |
| Cardiovascular diseases | 429 (47.5) | 1045 (36.0) | 942 (28.1) | 929 (20.4) | <0.001 |
| Diabetes | 106 (11.7) | 153 (5.3) | 89 (2.7) | 78 (1.7) | <0.001 |
| Long-standing illness | 484 (53.6) | 1149 (39.6) | 1110 (33.1) | 1220 (26.8) | <0.001 |
| **Grip strength (kg), mean (SD)** | 28.9 (10.9) | 29.9 (10.6) | 30.5 (10.5) | 30.1 (9.8) | <0.001 |
| **FEV1, (liter), mean (SD)** | 2.5 (0.7) | 2.6 (0.7) | 2.7 (0.7) | 2.8 (0.7) | <0.001 |

BMI, body mass index; FEV1, forced expiratory volume in 1 second; IQR, interquartile range; LPA, light-intensity physical activity; MPA, moderate -intensity physical activity; MVPA moderate to vigorous-intensity physical activity; SD standard deviation.

# Table S5. HRs and 95% CIs for mortality in participant groups divided by different levels of physical activity.

|  | **Time (minutes per week)** | **Participants** | **All-cause mortality** | **HRs (95% CI)** | **Cancer-specific mortality** | **HRs (95% CI)** |
| --- | --- | --- | --- | --- | --- | --- |
| **MVPA** | <272 | 2953 | 437 | ref | 266 | ref |
|  | 272-407 | 2905 | 226 | 0.64 (0.54, 0.76) | 155 | 0.71 (0.58, 0.88) |
|  | 407-579 | 2928 | 187 | 0.61 (0.51, 0.74) | 134 | 0.69 (0.55, 0.87) |
|  | ≥579 | 2922 | 133 | 0.52 (0.42, 0.66) | 101 | 0.61 (0.47, 0.81) |
| P for trend |  |  |  | **<0.001** |  | **<0.001** |
| **MPA** | <150 | 903 | 198 | ref | 106 | ref |
|  | 150-300 | 2905 | 307 | 0.57 (0.47, 0.70) | 204 | 0.74 (0.57, 0.95) |
|  | 300-450 | 3353 | 244 | 0.46 (0.37, 0.58) | 169 | 0.62 (0.47, 0.83) |
|  | ≥450 | 4547 | 235 | 0.41 (0.32, 0.53) | 177 | 0.59 (0.42, 0.81) |
| P for trend |  |  |  | **<0.001** |  | **0.005** |
| **LPA** | <1637 | 2939 | 347 | ref | 229 | ref |
|  | 1637-1939 | 2921 | 246 | 0.94 (0.79, 1.11) | 165 | 0.87 (0.70, 1.07) |
|  | 1939-2242 | 2932 | 206 | 0.91 (0.75, 1.09) | 140 | 0.81 (0.65, 1.01) |
|  | ≥2242 | 2916 | 184 | 0.94 (0.77, 1.16) | 122 | 0.79 (0.62, 1.01) |
| P for trend |  |  |  | **0.461** |  | **0.042** |

HRs and 95% CI for mortality in participant groups which were divided by different levels of physical activity were presented. All HRs and 95% CIs were calculated in multivariate Cox proportional hazards models that adjusted for LPA time/MVPA time/VPA time (continuous variable), age, sex, Townsend deprivation score, ethnicity, smoking status, alcohol intake frequency, BMI, waist circumference, self-reported general health, cancer duration, cardiovascular disease history, diabetes history, long-standing illness, disability or infirmity, grip strength, FEV1, and wear season. Linear trends were examined using the median value of each physical activity category as a continuous variable into the models.

# Table S6. Subgroup analysis between MVPA time (≥272 vs <272 minutes/week) and all-cause mortality.

| **Subgroups** | Participants | All-cause mortality | HRs (95% CI) | **P for interaction** |
| --- | --- | --- | --- | --- |
| **Age (years old)** |  |  |  | 0.601 |
| <65 | 4585 | 215 | 0.59 (0.42, 0.83) |  |
| ≥65 | 7123 | 768 | 0.62 (0.53, 0.74) |  |
| **Sex** |  |  |  | 0.881 |
| Female | 6923 | 438 | 0.68 (0.55, 0.86) |  |
| Male | 4785 | 545 | 0.56 (0.46, 0.68) |  |
| **BMI (kg/m^2^)** |  |  |  | 0.117 |
| <30 | 9517 | 729 | 0.58 (0.49, 0.69) |  |
| ≥30 | 2191 | 254 | 0.71 (0.53, 0.95) |  |
| **Waist** |  |  |  | 0.709 |
| Health | 8327 | 627 | 0.62 (0.52, 0.75) |  |
| Unhealth | 3381 | 356 | 0.58 (0.46, 0.75) |  |
| **Cancer type** |  |  |  | **0.021** |
| Malignant | 10123 | 931 | 0.61 (0.53, 0.72) |  |
| Non-malignant | 1585 | 52 | 0.54 (0.27, 1.08) |  |
| **Grip strength (kg)** |  |  |  | 0.429 |
| Low | 5626 | 544 | 0.64 (0.52, 0.78) |  |
| High | 6082 | 439 | 0.58 (0.47, 0.73) |  |
| **FEV1 (L)** |  |  |  | 0.436 |
| <2.63 | 5831 | 493 | 0.61 (0.50, 0.76) |  |
| ≥2.63 | 5877 | 490 | 0.62 (0.50, 0.76) |  |
| **Cancer duration (year)** |  |  |  | 0.971 |
| < 5 | 4278 | 412 | 0.59 (0.47, 0.75) |  |
| ≥5 | 7430 | 571 | 0.62 (0.51, 0.75) |  |

All HRs and 95% CIs were calculated in multivariate Cox proportional hazards models that adjusted for LPA time (continuous variable), age, sex, Townsend deprivation score, ethnicity, smoking status, alcohol intake frequency, BMI, waist circumference, self-reported general health, cancer duration, cardiovascular disease history, diabetes history, long-standing illness, disability or infirmity, grip strength, FEV1, and wear season. P values for interaction were calculated by likelihood ratio tests.

# Table S7. Subgroup analysis between MVPA time (≥272 vs <272 minutes/week) and cancer-specific mortality.

| **Subgroups** | Participants | Cancer-specific mortality | HRs (95% CI) | **P for interaction** |
| --- | --- | --- | --- | --- |
| **Age (years old)** |  |  |  | 0.557 |
| <65 | 4585 | 162 | 0.61 (0.41, 0.91) |  |
| ≥65 | 7123 | 494 | 0.71 (0.58, 0.88) |  |
| **Sex** |  |  |  | 0.548 |
| Female | 6923 | 311 | 0.72 (0.55, 0.95) |  |
| Male | 4785 | 345 | 0.66 (0.51,0.85) |  |
| **BMI (kg/m^2^)** |  |  |  | 0.436 |
| <30 | 9517 | 495 | 0.67 (0.54, 0.83) |  |
| ≥30 | 2191 | 161 | 0.74 (0.52, 1.07) |  |
| **Waist** |  |  |  | 0.948 |
| Health | 8327 | 431 | 0.69 (0.55, 0.87) |  |
| Unhealth | 3381 | 225 | 0.67 (0.49, 0.92) |  |
| **Cancer type** |  |  |  |  |
| Malignant | 10123 | 642 | 0.67 (0.56, 0.81) |  |
| Non-malignant | 1585 | 14 | NA |  |
| **Grip strength (kg)** |  |  |  | 0.398 |
| Low | 5626 | 340 | 0.74 (0.57, 0.96) |  |
| High | 6082 | 316 | 0.64 (0.49, 0.84) |  |
| **FEV1 (L)** |  |  |  | 0.343 |
| <2.63 | 5831 | 310 | 0.67 (0.51, 0.88) |  |
| ≥2.63 | 5877 | 346 | 0.71 (0.55, 0.92) |  |
| **Cancer duration (year)** |  |  |  | 0.991 |
| < 5 | 4278 | 283 | 0.66 (0.49, 0.88) |  |
| ≥5 | 7430 | 373 | 0.70 (0.55, 0.90) |  |

All HRs and 95% CIs were calculated in multivariate Cox proportional hazards models that adjusted for LPA time (continuous variable), age, sex, Townsend deprivation score, ethnicity, smoking status, alcohol intake frequency, BMI, waist circumference, self-reported general health, cancer duration, cardiovascular disease history, diabetes history, long-standing illness, disability or infirmity, grip strength, FEV1, and wear season. HR for cancer-specific mortality in non-malignant was not calculated due to the small number of events. P values for interaction were calculated by likelihood ratio tests.

# Table S8. Subgroup analysis between LPA time (≥1637 vs <1637 minutes/week) and all-cause mortality.

| **Subgroups** | Participants | All-cause mortality | HRs (95% CI) | **P for interaction** |
| --- | --- | --- | --- | --- |
| **Age (years old)** |  |  |  | 0.937 |
| <65 | 4585 | 215 | 0.97 (0.71, 1.34) |  |
| ≥65 | 7123 | 768 | 0.92 (0.78, 1.08) |  |
| **Sex** |  |  |  | 0.176 |
| Female | 6923 | 438 | **0.79 (0.63, 0.99)** |  |
| Male | 4785 | 545 | 1.02 (0.84, 1.23) |  |
| **BMI (kg/m^2^)** |  |  |  | 0.304 |
| <30 | 9517 | 729 | 0.88 (0.74, 1.05) |  |
| ≥30 | 2191 | 254 | 1.07 (0.80, 1.42) |  |
| **Waist** |  |  |  | 0.753 |
| Health | 8327 | 627 | 0.94 (0.78, 1.13) |  |
| Unhealth | 3381 | 356 | 0.95 (0.74, 1.20) |  |
| **Cancer type** |  |  |  | 0.113 |
| Malignant | 10123 | 931 | 0.94 (0.81, 1.10) |  |
| Non-malignant | 1585 | 52 | 0.82 (0.43, 1.55) |  |
| **Grip strength (kg)** |  |  |  | 0.243 |
| Low | 5626 | 544 | 1.03 (0.84, 1.26) |  |
| High | 6082 | 439 | 0.82 (0.67, 1.02) |  |
| **FEV1 (L)** |  |  |  | 0.274 |
| <2.63 | 5831 | 493 | 0.90 (0.73, 1.12) |  |
| ≥2.63 | 5877 | 490 | 0.96 (0.78, 1.17) |  |
| **Cancer duration (year)** |  |  |  | 0.202 |
| <5 | 4278 | 412 | 0.83 (0.67, 1.04) |  |
| ≥5 | 7430 | 571 | 1.02 (0.84, 1.24) |  |

All HRs and 95% CIs were calculated in multivariate Cox proportional hazards models that adjusted for MVPA time (continuous variable), age, sex, Townsend deprivation score, ethnicity, smoking status, alcohol intake frequency, BMI, waist circumference, self-reported general health, cancer duration, cardiovascular disease history, diabetes history, long-standing illness, disability or infirmity, grip strength, FEV1, and wear season. P values for interaction were calculated by likelihood ratio tests.

# Table S9. Subgroup analysis between LPA time (≥1637 vs <1637 minutes/week) and cancer-specific mortality.

| **Subgroups** | Participants | Cancer-specific mortality | HRs (95% CI) | **P for interaction** |
| --- | --- | --- | --- | --- |
| **Age (years old)** |  |  |  | 0.819 |
| <65 | 4585 | 162 | 0.87 (0.60, 1.25) |  |
| ≥65 | 7123 | 494 | 0.82 (0.67, 1.01) |  |
| **Sex** |  |  |  | 0.473 |
| Female | 6923 | 311 | 0.78 (0.59, 1.02) |  |
| Male | 4785 | 345 | 0.86 (0.68, 1.09) |  |
| **BMI (kg/m^2^)** |  |  |  | 0.721 |
| <30 | 9517 | 495 | 0.82 (0.67, 1.01) |  |
| ≥30 | 2191 | 161 | 0.86 (0.60, 1.23) |  |
| **Waist** |  |  |  | 0.896 |
| Health | 8327 | 431 | 0.83 (0.66, 1.04) |  |
| Unhealth | 3381 | 225 | 0.85 (0.63, 1.14) |  |
| **Cancer type** |  |  |  |  |
| Malignant | 10123 | 642 | 0.84 (0.70, 1.01) |  |
| Non-malignant | 1585 | 14 | NA |  |
| **Grip strength (kg)** |  |  |  | 0.502 |
| Low | 5626 | 340 | 1.03 (0.84, 1.26) |  |
| High | 6082 | 316 | 0.82 (0.67, 1.02) |  |
| **FEV1 (L)** |  |  |  | 0.319 |
| <2.63 | 5831 | 310 | 0.80 (0.61, 1.05) |  |
| ≥2.63 | 5877 | 346 | 0.86 (0.68, 1.10) |  |
| **Cancer duration (year)** |  |  |  | 0.135 |
| < 5 | 4278 | 283 | **0.72 (0.55, 0.94)** |  |
| ≥5 | 7430 | 373 | 0.94 (0.74, 1.20) |  |

All HRs and 95% CIs were calculated in multivariate Cox proportional hazards models that adjusted for MVPA time (continuous variable), age, sex, Townsend deprivation score, ethnicity, smoking status, alcohol intake frequency, BMI, waist circumference, self-reported general health, cancer duration, cardiovascular disease history, diabetes history, long-standing illness, disability or infirmity, grip strength, FEV1, and wear season. HR for cancer-specific mortality in non-malignant was not calculated due to the small number of events. P values for interaction were calculated by likelihood ratio tests.

# Table S10. Subgroup analysis between MVPA time (≥272 vs <272 minutes/week) and all-cause mortality in different sites.

| **Cancer type** | Participants | All-cause mortality | HRs (95% CI) | **P value** |
| --- | --- | --- | --- | --- |
| **Skin** | 1022 | 108 | ref |  |
|  | 3072 | 148 | 0.55 (0.41, 0.72) | **<0.001** |
| **Breast** | 499 | 64 | ref |  |
|  | 1664 | 84 | 0.47 (0.32, 0.68) | **<0.001** |
| **Genital** | 513 | 89 | ref |  |
|  | 1458 | 119 | 0.56 (0.41, 0.77) | **<0.001** |
| Male genital | 368  1098 | 65  92 | ref  0.54 (0.38 ,0.77) | **<0.001** |
| Female genital | 145  360 | 24  27 | ref  0.63 (0.34, 1.17) | 0.139 |
| **Digestive** | 243 | 61 | ref |  |
|  | 551 | 76 | 0.62 (0.43, 0.91) | **0.015** |
| **Blood** | 174 | 48 | ref |  |
|  | 365 | 46 | 0.50 (0.31, 0.81) | **0.004** |
| **Urinary** | 99  196 | 24  21 | ref  0.53 (0.27, 1.01) | 0.053 |
| **Lip, oral and pharynx**  **(head and neck)** | 37  85 | 7  11 | ref  0.72 (0.22, 2.39) | 0.594 |
| **Respiratory** | 40  61 | 17  9 | ref  0.48 (0.17, 1.33) | 0.155 |
| **Other** | 62 | 16 | ref |  |
|  | 187 | 13 | 0.46 (0.19, 1.14) | 0.093 |
| **Non-malignant** | 348 | 30 | ref |  |
|  | 1237 | 22 | 0.47 (0.24, 0.90) | **0.022** |

All HRs and 95% CIs were calculated in multivariate Cox proportional hazards models that adjusted for LPA time (continuous variable), age, sex and BMI. Error bars represent the 95% CIs.

# Table S11. Subgroup analysis between MVPA time (≥272 vs <272 minutes/week) and cancer-specific mortality in different sites.

| **Cancer type** | Participants | Cancer-specific mortality | HRs (95% CI) | **P value** |
| --- | --- | --- | --- | --- |
| **Skin** | 1022 | 44 | ref |  |
|  | 3072 | 94 | 0.87 (0.59, 1.31) | 0.512 |
| **Breast** | 499 | 47 | ref |  |
|  | 1664 | 73 | 0.52 (0.34, 0.79) | **0.002** |
| **Genital** | 513 | 66 | ref |  |
|  | 1458 | 83 | 0.55 (0.38, 0.80) | **0.002** |
| Male genital | 368  1098 | 46  60 | ref  0.53 (0.34, 0.82) | **0.004** |
| Female genital | 145  360 | 20  23 | ref  0.61 (0.31, 1.21) | 0.158 |
| **Digestive** | 243 | 44 | ref |  |
|  | 551 | 56 | 0.57 (0.38, 0.87) | **0.009** |
| **Blood** | 174 | 37 | ref |  |
|  | 365 | 30 | 0.41 (0.23, 0.72) | **0.002** |
| **Urinary** | 99  196 | 16  18 | ref  0.62 (0.29, 1.31) | 0.212 |
| **Lip, oral and pharynx**  **(head and neck)** | 37  85 | 5  7 | ref  0.67 (0.16, 2.82) | 0.589 |
| **Respiratory** | 40  61 | 10  8 | ref  0.89 (0.26, 3.03) | 0.855 |
| **Other** | 62  187 | 15  11 | ref  0.48 (0.18, 1.25) | 0.134 |
| **Non-malignant** | 348 | 5 | NA |  |
|  | 1237 | 9 | NA | NA |

All HRs and 95% CIs were calculated in multivariate Cox proportional hazards models that adjusted for LPA time (continuous variable), age, sex and BMI. Error bars represent the 95% CIs.

# Table S12. Subgroup analysis between LPA time (≥1637 vs <1637 minutes/week) and all-cause mortality in different sites.

| **Cancer type** | Participants | All-cause mortality | HRs (95% CI) | **P value** |
| --- | --- | --- | --- | --- |
| **Skin** | 1029 | 82 | ref |  |
|  | 3065 | 174 | 0.98 (0.74, 1.31) | 0.904 |
| **Breast** | 436 | 43 | ref |  |
|  | 1727 | 105 | 0.84 (0.57, 1.24) | 0.389 |
| **Genital** | 561 | 68 | ref |  |
|  | 1410 | 140 | 1.14 (0.83, 1.57) | 0.403 |
| Male genital | 457  1009 | 56  101 | ref  1.13 (0.79, 1.61) | 0.500 |
| Female genital | 104  401 | 12  39 | ref  1.17 (0.58, 2.36) | 0.665 |
| **Digestive** | 218 | 56 | ref |  |
|  | 576 | 81 | 0.72 (0.49, 1.04) | 0.083 |
| **Blood** | 169 | 32 | ref |  |
|  | 370 | 62 | 1.07 (0.69, 1.64) | 0.774 |
| **Urinary** | 89  206 | 20  25 | ref  0.67 (0.35, 1.29) | 0.232 |
| **Lip, oral and pharynx**  **(head and neck)** | 32  90 | 6  12 | ref  1.18 (0.37, 3.80) | 0.784 |
| **Respiratory** | 39  62 | 14  12 | ref  0.86 (0.36, 2.03) | 0.728 |
| **Other** | 67  182 | 13  16 | ref  0.62 (0.27, 1.39) | 0.244 |
| **Non-malignant** | 360 | 22 | ref |  |
|  | 1225 | 30 | 0.73 (0.39, 1.36) | 0.327 |

All HRs and 95% CIs were calculated in multivariate Cox proportional hazards models that adjusted for MVPA time (continuous variable), age, sex and BMI. Error bars represent the 95% CIs.

# Table S13. Subgroup analysis between LPA time (≥1637 vs <1637 minutes/week) and cancer-specific mortality in different sites.

| **Cancer type** | Participants | Cancer-specific mortality | HRs (95% CI) | **P value** |
| --- | --- | --- | --- | --- |
| **Skin** | 1029 | 42 | ref |  |
|  | 3065 | 96 | 0.88 (0.59, 1.31) | 0.524 |
| **Breast** | 436 | 32 | ref |  |
|  | 1727 | 88 | 0.88 (0.56, 1.36) | 0.559 |
| **Genital** | 561 | 49 | ref |  |
|  | 1410 | 100 | 1.07 (0.73, 1.55) | 0.733 |
| Male genital | 457  1009 | 39  67 | ref  1.04 (0.68, 1.59) | 0.866 |
| Female genital | 104  401 | 10  33 | ref  1.15 (0.53 ,2.47) | 0.723 |
| **Digestive** | 218 | 42 | ref |  |
|  | 576 | 58 | 0.65 (0.42, 1.00) | 0.052 |
| **Blood** | 169 | 26 | ref |  |
|  | 370 | 41 | 1.45 (0.84, 2.50) | 0.187 |
| **Urinary** | 89  206 | 13  21 | ref  0.81 (0.38, 1.72) | 0.578 |
| **Lip, oral and pharynx**  **(head and neck)** | 32  90 | 4  8 | ref  0.79 (0.18, 3.47) | 0.757 |
| **Respiratory** | 39  62 | 9  9 | ref  0.93 (0.33, 2.63) | 0.897 |
| **Other** | 67  182 | 12  14 | ref  0.57 (0.24, 1.35) | 0.202 |
| **Non-malignant** | 360 | 5 | NA |  |
|  | 1225 | 9 | NA | NA |

All HRs and 95% CIs were calculated in multivariate Cox proportional hazards models that adjusted for MVPA time (continuous variable), age, sex and BMI. Error bars represent the 95% CIs.

# Table S14. Methods of sensitivity analysis and outcome event summary.

|  | **Method** | **Participants** | **Outcome** | | |
| --- | --- | --- | --- | --- | --- |
|  |  |  | **All-cause** | **Cancer-specific, n (%)** | **CVD, n (%)** |
| Sensitivity analysis 1 | Exclude:  Non-malignant  +  Poor self-reported medical condition | 9823 | 868 | 609 (70.2%) | 117 (13.5%) |
| Sensitivity analysis 2 | Exclude:  Non-malignant  +  Poor self-reported medical condition  +  Long-standing illness or disability | 6595 | 483 | 374 (77.4%) | 54 (11.2%) |
| Sensitivity analysis 2 | Exclude:  Non-malignant  +  Poor self-reported medical condition  +  Long-standing illness or disability  +  Died within two years since wearing accelerometer | 6503 | 397 | 295 (74.3%) | 47 (11.8%) |

# Table S15. Baseline characteristics of participants by quartiles of MVPA time after excluding non-malignance and those with poor self-reported medical condition (n=9823).

|  | Quartiles of MVPA time (minutes/week) | | | | P value |  |
| --- | --- | --- | --- | --- | --- | --- |
|  | <272 | 272-407 | 407-579 | ≥579 |  |  |
| n | 2458 | 2457 | 2495 | 2413 |  |  |
| **Age (years), mean (SD)** | 68.5 (5.8) | 66.7 (6.5) | 65.4 (6.8) | 63.5 (7.1) | <0.001 |  |
| **Male, n (%)** | 1152 (46.9) | 1104 (44.9) | 1077 (43.2) | 977 (40.5) | <0.001 |  |
| **Townsend deprivation score, mean (SD)** | -2.0 (2.7) | -2.0 (2.6) | -2.1 (2.6) | -2.0 (2.6) | 0.54 |  |
| **Ethnicity, n (%)** |  |  |  |  |  |  |
| White | 2424 (98.6) | 2414 (98.2) | 2455 (98.4) | 2361 (97.8) |  |  |
| Asian | 8 (0.3) | 15 (0.6) | 12 (0.5) | 10 (0.4) |  |  |
| Blacks | 7 (0.3) | 4 (0.2) | 10 (0.4) | 6 (0.2) |  |  |
| Mixed | 5 (0.2) | 7 (0.3) | 7 (0.3) | 13 (0.5) |  |  |
| Other | 14 (0.6) | 17 (0.7) | 11 (0.4) | 23 (1.0) |  |  |
| **Smoking status, n (%)** |  |  |  |  | <0.001 |  |
| Current | 200 (8.1) | 146 (5.9) | 113 (4.5) | 105 (4.4) |  |  |
| Previous | 1044 (42.5) | 956 (38.9) | 961 (38.5) | 916 (38.0) |  |  |
| Never | 1214 (49.4) | 1355 (55.1) | 1421 (57.0) | 1392 (57.7) |  |  |
| **Alcohol intake frequency, n (%)** |  |  |  |  | <0.001 |  |
| Daily | 598 (24.3) | 622 (25.3) | 634 (25.4) | 569 (23.6) |  |  |
| 3-4 times/week | 586 (23.8) | 622 (25.3) | 709 (28.4) | 732 (30.3) |  |  |
| 1-2 times/week | 587 (23.9) | 590 (24.0) | 610 (24.4) | 591 (24.5) |  |  |
| 1-3 times/month | 261 (10.6) | 250 (10.2) | 232 (9.3) | 236 (9.8) |  |  |
| Social drinking or never | 426 (17.3) | 373 (15.2) | 310 (12.4) | 285 (11.8) |  |  |
| **BMI (kg/m^2^), mean (SD)** | 28.1 (4.7) | 26.8 (4.3) | 26.15 (3.99) | 25.1 (3.6) | <0.001 |  |
| **Waist circumference (cm), mean (SD)** | 93.2 (13.2) | 89.5 (12.5) | 87.3 (11.8) | 83.9 (11.4) | <0.001 |  |
| **LPA (minute), mean (SD)** | 1632.1 (436.8) | 1903.9 (405.0) | 2038.0 (397.2) | 2188.88 (399.5) | <0.001 |  |
| **MVPA (minute), mean (SD)** | 184.9 (62.9) | 340.7 (38.2) | 487.1 (49.0) | 763.8 (168.6) | <0.001 |  |
| **Self-reported general health, n (%)** |  |  |  |  | <0.001 |  |
| Excellent | 321 (13.1) | 410 (16.7) | 537 (21.5) | 618 (25.6) |  |  |
| Good | 1517 (61.7) | 1531 (62.3) | 1587 (63.6) | 1509 (62.5) |  |  |
| Fair | 620 (25.2) | 516 (21.0) | 371 (14.9) | 286 (11.9) |  |  |
| **Cancer duration (year), median (IQR)** | 6.8 (2.9, 12.2) | 6.8 (3.0, 12.4) | 6.5 (3.0, 12.1) | 7.1 (3.1, 12.6) | 0.247 |  |
| **Other medical history, n (%)** |  |  |  |  |  |  |
| Cardiovascular diseases | 955 (38.9) | 748 (30.4) | 651 (26.1) | 442 (18.3) |  |  |
| Diabetes | 168 (6.8) | 69 (2.8) | 39 (1.6) | 36 (1.5) | <0.001 |  |
| Long-standing illness | 1048 (42.6) | 850 (34.6) | 712 (28.5) | 618 (25.6) | <0.001 |  |
| **Grip strength (kg), mean (SD)** | 30.0 (10.8) | 30.5 (10.6) | 30.9 (10.4) | 30.8 (9.9) | 0.014 |  |
| **FEV1, (liter), mean (SD)** | 2.6 (0.7) | 2.7 (0.7) | 2.8 (0.7) | 2.8 (0.7) | <0.001 |  |

BMI, body mass index; FEV1, forced expiratory volume in 1 second; IQR, interquartile range; LPA, light-intensity physical activity; MVPA moderate to vigorous-intensity physical activity; SD standard deviation.

# Table S16. Baseline characteristics of participants by quartiles of MVPA time after excluding non-malignance, those with poor self-reported medical condition and long-standing illness (n=6595).

|  | Quartiles of MVPA time (minutes/week) | | | | P value |  |
| --- | --- | --- | --- | --- | --- | --- |
|  | <272 | 272-407 | 407-579 | ≥579 |  |  |
| n | 1410 | 1607 | 1783 | 1795 |  |  |
| **Age (years), mean (SD)** | 68.2 (6.0) | 66.5 (6.6) | 65.4 (6.8) | 63.3 (7.1) | <0.001 |  |
| **Male, n (%)** | 625 (44.3) | 677 (42.1) | 751 (42.1) | 708 (39.4) | <0.001 |  |
| **Townsend deprivation score, mean (SD)** | -2.1 (2.7) | -2.2 (2.5) | -2.2 (2.6) | -2.0 (2.6) | 0.046 |  |
| **Ethnicity, n (%)** |  |  |  |  |  |  |
| White | 1398 (99.1) | 1582 (98.4) | 1757 (98.5) | 1760 (98.1) |  |  |
| Asian | 2 (0.1) | 7 (0.4) | 6 (0.3) | 7 (0.4) |  |  |
| Blacks | 2 (0.1) | 2 (0.1) | 6 (0.3) | 5 (0.3) |  |  |
| Mixed | 3 (0.2) | 6 (0.4) | 6 (0.3) | 8 (0.4) |  |  |
| Other | 5 (0.4) | 10 (0.6) | 8 (0.4) | 15 (0.8) |  |  |
| **Smoking status, n (%)** |  |  |  |  | <0.001 |  |
| Current | 106 (7.5) | 92 (5.7) | 76 (4.3) | 82 (4.6) |  |  |
| Previous | 570 (40.4) | 616 (38.3) | 666 (37.4) | 669 (37.3) |  |  |
| Never | 734 (52.1) | 899 (55.9) | 1041 (58.4) | 1044 (58.2) |  |  |
| **Alcohol intake frequency, n (%)** |  |  |  |  | <0.001 |  |
| Daily | 364 (25.8) | 423 (26.3) | 464 (26.0) | 426 (23.7) |  |  |
| 3-4 times/week | 347 (24.6) | 429 (26.7) | 528 (29.6) | 563 (31.4) |  |  |
| 1-2 times/week | 341 (24.2) | 381 (23.7) | 447 (25.1) | 449 (25.0) |  |  |
| 1-3 times/month | 155 (11.0) | 163 (10.1) | 150 (8.4) | 167 (9.3) |  |  |
| Social drinking or never | 203 (14.4) | 211 (13.1) | 194 (10.9) | 190 (10.6) |  |  |
| **BMI (kg/m^2^), mean (SD)** | 27.6 (4.4) | 26.5 (4.1) | 25.9 (3.8) | 25.0 (3.5) | <0.001 |  |
| **Waist circumference (cm), mean (SD)** | 91.4 (12.4) | 88.2 (12.1) | 86.5 (11.4) | 83.3 (11.1) | <0.001 |  |
| **LPA (minute), mean (SD)** | 1660.8 (433.3) | 1899.8 (402.6) | 2040.5 (398.9) | 2188.4 (398.7) | <0.001 |  |
| **MVPA (minute), mean (SD)** | 191.8 (60.9) | 341.2 (38.2) | 487.6 (49.2) | 766.2 (168.8) | <0.001 |  |
| **Self-reported general health, n (%)** |  |  |  |  | <0.001 |  |
| Excellent | 265 (18.8) | 365 (22.7) | 479 (26.9) | 552 (30.8) |  |  |
| Good | 971 (68.9) | 1068 (66.5) | 1167 (65.5) | 1128 (62.8) |  |  |
| Fair | 174 (12.3) | 174 (10.8) | 137 (7.7) | 115 (6.4) |  |  |
| **Cancer duration (year), median (IQR)** | 6.1 (2.7, 11.9) | 6.7 (2.8, 12.4) | 6.0 (2.7, 11.9) | 6.6 (3.0, 12.6) | 0.062 |  |
| **Other medical history, n (%)** |  |  |  |  |  |  |
| Cardiovascular diseases | 462 (32.8) | 411 (25.6) | 391 (21.9) | 277 (15.4) | <0.001 |  |
| Diabetes | 27 (1.9) | 8 (0.5) | 10 (0.6) | 10 (0.6) | <0.001 |  |
| **Grip strength (kg), mean (SD)** | 30.2 (10.7) | 30.6 (10.4) | 31.0 (10.3) | 30.9 (9.7) | 0.095 |  |
| **FEV1, (liter), mean (SD)** | 2.7 (0.7) | 2.8 (0.7) | 2.8 (0.7) | 2.9 (0.7) | <0.001 |  |

BMI, body mass index; FEV1, forced expiratory volume in 1 second; IQR, interquartile range; LPA, light-intensity physical activity; MVPA moderate to vigorous-intensity physical activity; SD standard deviation.

# Table S17. Baseline characteristics of participants by quartiles of MVPA time after excluding non-malignance, those with poor self-reported medical condition, with long-standing illness, and died within two years since wearing accelerometer (n=6503).

|  | Quartiles of MVPA time (minutes/week) | | | | P value |  |
| --- | --- | --- | --- | --- | --- | --- |
|  | <272 | 272-407 | 407-579 | ≥579 |  |  |
| n | 1388 | 1578 | 1764 | 1773 |  |  |
| **Age (years), mean (SD)** | 68.1 (6.0) | 66.3 (6.6) | 65.2 (6.8) | 63.2 (7.1) | <0.001 |  |
| **Male, n (%)** | 606 (43.7%) | 660 (41.8%) | 744 (42.2%) | 697 (39.3%) | 0.089 |  |
| **Townsend deprivation score, mean (SD)** | -2.1 (2.7) | -2.2 (2.5) | -2.2 (2.6) | -2.0 (2.6) | 0.006 |  |
| **Ethnicity, n (%)** |  |  |  |  |  |  |
| White | 1377 (99.2) | 1557 (98.7) | 1741 (98.7) | 1747 (98.5) |  |  |
| Asian | 2 (0.1) | 7 (0.4) | 6 (0.3) | 5 (0.3) |  |  |
| Blacks | 2 (0.1) | 2 (0.1) | 5 (0.3) | 5 (0.3) |  |  |
| Mixed | 3 (0.2) | 4 (0.3) | 5 (0.3) | 3 (0.2) |  |  |
| Other | 4 (0.3) | 8 (0.5) | 7 (0.4) | 13 (0.7) |  |  |
| **Smoking status, n (%)** |  |  |  |  | <0.001 |  |
| Current | 101 (7.3%) | 90 (5.7%) | 75 (4.3%) | 81 (4.6%) |  |  |
| Previous | 559 (40.3%) | 599 (38.0%) | 660 (37.4%) | 659 (37.2%) |  |  |
| Never | 728 (52.4%) | 889 (56.3%) | 1029 (58.3%) | 1033 (58.3%) |  |  |
| **Alcohol intake frequency, n (%)** |  |  |  |  | <0.001 |  |
| Daily | 353 (25.4%) | 416 (26.4%) | 455(25.8%) | 422 (23.8%) |  |  |
| 3-4 times/week | 338 (24.4%) | 421 (26.7%) | 523 (29.6%) | 559 (31.5%) |  |  |
| 1-2 times/week | 340 (24.5%) | 374 (23.7%) | 440 (24.9%) | 443 (25.0%) |  |  |
| 1-3 times/month | 155 (11.2%) | 160 (10.1%) | 150 (8.5%) | 164 (9.2%) |  |  |
| Social drinking or never | 202 (14.6%) | 207 (13.1%) | 196(11.1%) | 185 (10.4%) |  |  |
| **BMI (kg/m^2^), mean (SD)** | 27.6 (4.4) | 26.5 (4.1) | 25.9 (3.8) | 24.9 (3.4) | <0.001 |  |
| **Waist circumference (cm), mean (SD)** | 91.2 (12.4) | 88.2 (12.1) | 86.5 (11.5) | 83.3 (11.0) | <0.001 |  |
| **LPA (minute), mean (SD)** | 1669.5 (429.5) | 1901.7 (403.3) | 2041.7 (399.5) | 2188.1 (400.0) | <0.001 |  |
| **MVPA (minute), mean (SD)** | 194.4 (60.2) | 342.6 (38.0) | 488.9 (49.2) | 766.9 (168.8) | <0.001 |  |
| **Self-reported general health, n (%)** |  |  |  |  | <0.001 |  |
| Excellent | 264 (19.0%) | 361 (22.9%) | 469 (26.6%) | 548 (30.9%) |  |  |
| Good | 953 (68.7%) | 1048 (66.4%) | 1160 (65.8%) | 1112 (62.7%) |  |  |
| Fair | 171 (12.3%) | 169 (10.7%) | 135 (7.7%) | 113 (6.4%) |  |  |
| **Cancer duration (year), median (IQR)** | 8.3 (7.4) | 8.7 (7.7) | 8.1 (7.2) | 8.6 (7.2) | 0.080 |  |
| **Other medical history, n (%)** |  |  |  |  |  |  |
| Cardiovascular diseases | 452 (32.6%) | 399 (25.3%) | 391 (22.2%) | 272 (15.3%) | <0.001 |  |
| Diabetes | 23 (1.7%) | 7 (0.4%) | 9 (0.5%) | 10 (0.6%) | <0.001 |  |
| **Grip strength (kg), mean (SD)** | 30.1 (10.7) | 30.6 (10.4) | 31.0 (10.3) | 30.9 (9.7) | 0.014 |  |
| **FEV1, (liter), mean (SD)** | 2.6 (0.7) | 2.7 (0.7) | 2.8 (0.7) | 2.8 (0.7) | <0.001 |  |

BMI, body mass index; FEV1, forced expiratory volume in 1 second; IQR, interquartile range; LPA, light-intensity physical activity; MVPA moderate to vigorous-intensity physical activity; SD standard deviation.

# Table S18. Cox regression results of sensitivity analysis that excluded non-malignant participants and those with poor self-reported medical condition.

|  | Time (minutes per week) | Participants | All-cause death | HRs (95% CI) | Cancer-specific death | HRs (95% CI) |
| --- | --- | --- | --- | --- | --- | --- |
| **MVPA** | <272 | 2458 | 367 | ref | 243 | ref |
|  | 272-407 | 2457 | 209 | 0.67 (0.56, 0.80) | 144 | 0.69 (0.55, 0.86) |
|  | 407-579 | 2495 | 170 | 0.61 (0.50, 0.74) | 127 | 0.66 (0.52, 0.84) |
|  | ≥579 | 2413 | 122 | 0.52 (0.41, 0.66) | 95 | 0.58 (0.43, 0.76) |
| **LPA** | <1637 | 2443 | 294 | ref | 206 | ref |
|  | 1637-1939 | 2494 | 222 | 0.94 (0.78, 1.12) | 153 | 0.86 (0.69, 1.06) |
|  | 1939-2242 | 2455 | 184 | 0.91 (0.75, 1.11) | 132 | 0.83 (0.65, 1.05) |
|  | ≥2242 | 2431 | 168 | 0.96 (0.78, 1.20) | 118 | 0.83 (0.64, 1.07) |
| **MPA** | <150 | 727 | 156 | ref | 92 | ref |
|  | 150-300 | 2466 | 275 | 0.61 (0.49, 0.75) | 193 | 0.74 (0.57, 0.97) |
|  | 300-450 | 2824 | 221 | 0.49 (0.38, 0.62) | 155 | 0.59 (0.44, 0.80) |
|  | ≥450 | 3806 | 216 | 0.42 (0.32, 0.56) | 169 | 0.56 (0.40, 0.78) |

HRs and 95% CI for mortality in participant groups which were divided by different levels of physical activity were presented. All HRs and 95% CIs were calculated in multivariate Cox proportional hazards models that adjusted for LPA time/MVPA time/VPA time (continuous variable), age, sex, Townsend deprivation score, ethnicity, smoking status, alcohol intake frequency, BMI, waist circumference, self-reported general health, cancer duration, cardiovascular disease history, diabetes history, long-standing illness, disability or infirmity, grip strength, FEV1 and wear season.

# Table S19. Cox regression results of sensitivity analysis that excluded non-malignant participants, those with poor self-reported medical condition and long-standing illness.

|  | Time (minutes per week) | Participants | All-cause death | HRs (95% CI) | Cancer-specific death | HRs (95% CI) |
| --- | --- | --- | --- | --- | --- | --- |
| **MVPA** | <272 | 1410 | 175 | ref | 132 | ref |
|  | 272-407 | 1607 | 109 | 0.63 (0.49 ,0.81) | 84 | 0.63 (0.48, 0.84) |
|  | 407-579 | 1783 | 121 | 0.69 (0.54, 0.90) | 93 | 0.69 (0.51, 0.92) |
|  | ≥579 | 1795 | 78 | 0.51 (0.38 ,0.70) | 65 | 0.54 (0.38 ,0.76) |
| **LPA** | <1637 | 1488 | 144 | ref | 113 | ref |
|  | 1637-1939 | 1685 | 119 | 0.88 (0.68, 1.13) | 93 | 0.84 (0.63, 1.11) |
|  | 1939-2242 | 1692 | 120 | 0.99 (0.76, 1.28) | 94 | 0.92 (0.69, 1.23) |
|  | ≥2242 | 1730 | 100 | 0.91 (0.68, 1.21) | 74 | 0.79 (0.57, 1.09) |
| **MPA** | <150 | 356 | 64 | ref | 43 | ref |
|  | 150-300 | 1539 | 143 | 0.61 (0.45, 0.84) | 111 | 0.73 (0.50, 1.05) |
|  | 300-450 | 1903 | 133 | 0.53 (0.38, 0.74) | 100 | 0.61 (0.41, 0.92) |
|  | ≥450 | 2797 | 143 | 0.46 (0.31, 0.67) | 120 | 0.60 (0.39, 0.93) |

HRs and 95% CI for mortality in participant groups which were divided by different levels of physical activity were presented. All HRs and 95% CIs were calculated in multivariate Cox proportional hazards models that adjusted for LPA time/MVPA time/VPA time (continuous variable), age, sex, Townsend deprivation score, ethnicity, smoking status, alcohol intake frequency, BMI, waist circumference, self-reported general health, cancer duration, cardiovascular disease history, diabetes history, grip strength, FEV1 and wear season.

# Table S20. Cox regression results of sensitivity analysis that excluded non-malignant participants, those with poor self-reported medical condition, with long-standing illness, and died within two years since wearing accelerometer.

|  | Time (minutes per week) | Participants | All-cause death | HRs (95% CI) | Cancer-specific death | HRs (95% CI) |
| --- | --- | --- | --- | --- | --- | --- |
| **MVPA** | <272 | 1388 | 135 | ref | 94 | ref |
|  | 272-407 | 1578 | 187 | 0.66 (0.50 ,0.87) | 63 | 0.66 (0.48, 0.93) |
|  | 407-579 | 1764 | 112 | 0.83 (0.63, 1.09) | 86 | 0.88 (0.64, 1.22) |
|  | ≥579 | 1773 | 63 | 0.52 (0.37 ,0.74) | 52 | 0.59 (0.40 ,0.87) |
| **LPA** | <1637 | 1450 | 110 | ref | 83 | ref |
|  | 1637-1939 | 1665 | 98 | 0.91 (0.69, 1.21) | 74 | 0.86 (0.62, 1.19) |
|  | 1939-2242 | 1673 | 104 | 1.10 (0.82, 1.46) | 77 | 0.97 (0.70, 1.36) |
|  | ≥2242 | 1715 | 85 | 0.96 (0.70, 1.32) | 61 | 0.80 (0.55, 1.16) |
| **MPA** | <150 | 331 | 43 | ref | 24 | ref |
|  | 150-300 | 1509 | 116 | 0.72 (0.50, 1.04) | 87 | 0.87 (0.55, 1.38) |
|  | 300-450 | 1869 | 113 | 0.64 (0.43, 0.95) | 80 | 0.67 (0.42, 1.08) |
|  | ≥450 | 2794 | 125 | 0.54 (0.36, 0.82) | 104 | 0.60 (0.37, 0.96) |

HRs and 95% CI for mortality in participant groups which were divided by different levels of physical activity were presented. All HRs and 95% CIs were calculated in multivariate Cox proportional hazards models that adjusted for LPA time/MVPA time/VPA time (continuous variable), age, sex, Townsend deprivation score, ethnicity, smoking status, alcohol intake frequency, BMI, waist circumference, self-reported general health, cancer duration, cardiovascular disease history, diabetes history, grip strength, FEV1 and wear season.
